# Environmental conditions and ecological interactions among microorganisms isolated from diseased samples shape their biocontrol efficacy against *Erwinia amylovora*

**DOI:** 10.3389/fmicb.2026.1816023

**Published:** 2026-04-10

**Authors:** Ricardo Delgado Santander, Youfu Zhao

**Affiliations:** Department of Plant Pathology, College of Agricultural, Human, and Natural Resource Sciences, Irrigated Agriculture Research and Extension Center, Washington State University, Prosser, WA, United States

**Keywords:** antagonism, apple, fire blight, microbial interactions, synergism

## Abstract

**Introduction:**

Fire blight, caused by *Erwinia amylovora*, is a devastating disease of apples and pears. Limitations in current control strategies using antibiotics and copper demand for more sustainable alternatives . This study aimed to evaluate the potential of 13 microorganisms (12 bacteria, 1 yeast) co-isolated with *E. amylovora* from symptomatic apple tissues as biocontrol agents (BCAs) and determine how environmental conditions and ecological interactions influence their efficacy against the pathogen.

**Methods:**

We optimized an *in vitro* agar plug assay to determine antagonistic activities of microorganisms under different environmental conditions. We utilized *ex vivo* assays on detached fruitlets to determine the efficacies of BACs. Bliss independence and best single agent frameworks were used to identify BAC helpers and hinderers.

**Results:**

*In vitro* agar plug assays revealed that antagonistic activity of these microbes was enhanced by environmental factors, including minimal media, acidic pH (6.0), lower temperature (22°C), and specific carbon sources (fructose and glucose), which significantly enhanced their pathogen inhibition abilities. *Ex vivo* assays on detached fruitlets demonstrated that preventive application of BCAs was more effective at reducing disease symptoms than a co-inoculation (curative) regime. Most combinations of antagonists reduced infection rates, with combinations containing Erwinia sp. 2186 achieving the highest disease suppression. Net interaction analysis using Bliss independence and best single agent frameworks identified biocontrol helper and hinderer organisms. As an example, *Rahnella* sp. EL51 and *Pseudomonas* sp. 2180 acted to synergistically enhance or antagonistically decrease disease suppression in paired treatments, respectively. Population dynamics of the top-performing pair (*Erwinia* sp. 2186 plus *Rahnella* sp. EL51) indicated a link between disease suppression, sustained BCA presence, bacteriostatic effects on *E. amylovora* populations and medium acidification.

**Conclusion:**

These findings underscore the importance of ecological interactions and environmental effects in developing biocontrol strategies against *E. amylovora*.

## 1 Introduction

Fire blight, caused by the Gram-negative pathogen *Erwinia amylovora* (Burrill) (Winslow et al., 1920), is one of the bacterial diseases with major economic impact on apple and pear production worldwide (Aćimović et al., 2023; Nieto et al., 2024; Gusberti et al., 2015; Rickard et al., 2023; Sun et al., 2023; Zhao et al., 2019). The pathogen preferably infects blossoms and actively growing green tissue, although every host plant organ is susceptible to the pathogen. The main symptoms are expanding necrotic lesions, ooze production, and canker formation in perennial tissues and, under favorable conditions, infections can lead to the destruction of an entire orchard in a single season (Thomson, 2000; van der Zwet et al., 2016b).

Current fire blight management relies heavily on copper-based bactericides and antibiotics (streptomycin, kasugamycin and oxytetracycline), but their use is increasingly facing scrutiny due to the rise of antibiotic-resistant strains (Loper et al., 1991; Schmidt et al., 2025; Sundin et al., 2023), regulatory constraints, and social pressure with a trend toward more environmentally friendly disease control strategies (Sundin et al., 2016). As sustainable alternatives, biocontrol agents (BCAs) have shown promise, with bacterial and yeast strains of the genera *Pantoea*, *Pseudomonas*, *Bacillus*, and *Aureobasidium*, demonstrating efficacy in suppressing fire blight disease (Cabrefiga et al., 2007; Medhioub et al., 2022; Mikiciński et al., 2020; Pusey et al., 2009; Stockwell et al., 2002, 2010, 2011; Stockwell and Stack, 2007). However, inconsistent field performance in different regions, limited understanding of ecological interactions among BCAs and pathogens, and variable adaptation and persistence in different hosts and environmental conditions hinder their widespread adoption (Bonaterra et al., 2012, 2022).

The ecological niche of *E. amylovora* within infected plant tissues is shared with a diverse microbial community (Kong et al., 2021; Lee et al., 2023), which may compete for resources or produce inhibitory compounds. These co-inhabiting microorganisms represent an underexplored reservoir of potential BCAs naturally adapted to the host environment, which may possess specialized antagonistic mechanisms against *E. amylovora* as demonstrated in different pathosystems (Anees et al., 2010; Biosca et al., 2024; Huang et al., 2013; Tolba and Soliman, 2013). The most common antagonism strategies observed in BCAs are: (i) preemptive exclusion, where microorganisms compete for nutrients and space, (ii) production of antimicrobial compounds (antibiotics, bacteriocins, lipopeptides), (iii) contact-dependent inhibition (via type VI secretion systems or other systems); (iv) hyperparasitism and predation, and (v) induction or priming of plant defenses (Köhl et al., 2019; Peterson et al., 2020). However, biocontrol efficacy is highly context-dependent, influenced by environmental conditions (temperature, relative humidity), pathosystem factors (host species, pathogen strain, plant organ/tissue, nutrient composition and availability, pH), as well as BCA compatibility with the host plant microbiota and other BCAs when applied in mixes (Bonaterra et al., 2022; Cabrefiga et al., 2011; Hagen et al., 2009; Legein et al., 2020; Pujol et al., 2006; Pusey et al., 2011; Stockwell et al., 2011; Xu et al., 2011; Xu and Jeger, 2013a, b).

*In vitro* assays are critical for initial BCA screening but often fail to predict *in planta* performance due to oversimplified conditions and biased results toward certain pathogen antagonism mechanisms over others (Hossain, 2024). For example, nutrient-rich media may mask antagonistic traits linked to nutrient competition; carbon sources in the media, pH or incubation temperatures may affect antimicrobial metabolite production; and efficacious plant defense-inducing BCAs may be discarded through initial *in vitro* screenings in culture media (Besset-Manzoni et al., 2019). Besides, *in vitro* conditions usually do not reproduce physicochemical changes occurring within host tissues, such as changes in pH, tissue collapse or nutrient leakage during infections, or BCA and pathogen interactions with other microorganisms inhabiting the same microhabitat. Thus, optimizing *in vitro* assay conditions to mimic the host environment could improve the selection of robust BCAs.

Beyond single-strain applications, combining BCAs may enhance disease control through additive or synergistic effects (Guetsky et al., 2002; Xu et al., 2011), improve the survival and persistence of BCAs in different environments, and complement disease suppression with other properties, such as plant growth promoting activity (Cui et al., 2021; Nunes et al., 2024). Yet, net interactions between BCAs are poorly documented, and empirical frameworks to evaluate strain compatibility are rarely applied in phytopathology (Pandit et al., 2022; Xu et al., 2011). Detached fruit or flower assays offer a bridge between *in vitro* and field studies, enabling controlled investigation of net BCA interactions while accounting for host-specific factors.

We hypothesized that apple-associated microorganisms co-isolated with *E. amylovora* from fire blight lesions may possess strain-specific and combinatorial potential to antagonize the pathogen and suppress disease development. In this work, we characterized a collection of 13 microorganisms isolated from necrosed apple tissues, including Gram-positive and Gram-negative bacteria and one yeast. We first optimized an agar plug assay and tested varying nutritional, pH and incubation temperature conditions for improving detection of *E. amylovora* growth inhibition. We evaluated the potential of these 13 BCA candidates to reduce symptom severity and disease incidence using *ex vivo* assays with detached pears and apples. We tested the efficacy of single and paired isolate combinations in suppressing disease and used Bliss independence and “best single agent” frameworks to determine net interactions between paired-BCAs and their contribution to disease suppression. Finally, we analyzed population dynamics and effects on media pH by the top performing BCA mix. By integrating *in vitro* and *ex vivo* approaches, this study provides proof of concept for leveraging BCA interactions in fire blight management, while highlighting key parameters for future field validation.

## 2 Materials and methods

### 2.1 Microorganism isolation and culture conditions

Microorganism isolates, including six Gram-positive, six Gram-negative bacteria and one yeast, were co-isolated with *E. amylovora* from symptomatic apple tree samples collected in 2022 from commercial orchards in Benton, Franklin, and Yakima Counties in Washington State, U.S.A. (Table 1). Plant material was macerated in PBS and streaked in parallel on Crystal violet-Cycloheximide-Thallium nitrate (CCT) agar (Ishimaru and Klos, 1984) and Lysogeny agar (LA) (Bertani, 1951) to isolate *E. amylovora* and other microorganisms, respectively. Bacterial and yeast isolates were identified through partial sequencing of 16S rDNA using primers fD1/rP2 (Weisburg et al., 1991) and 26S rDNA using primers NL1/NL4 (Kurtzman and Robnett, 1997), respectively.

**TABLE 1:** Microorganisms co-isolated with *E. amylovora* from symptomatic apple tissues: source, identity and phenotypes.

				Activity halo diameter (mm ± SD)^bc^			
Isolate ID	Sample/host	Location	Identification^a^	Amylase	Protease	Lipase	% SU ± SD
Gram-positive							
2173	Necrosed leaf/“Gala” apple tree	Kennewick, WA	*Microbacterium* sp.	2.8 ± 0.6 **cd**	UA **e**	UA **d**	43.1 ± 0.5 **ce**
ABR10	Necrosed leaf/“Envy” apple tree	Othello, WA	*Microbacterium* sp.	UA **e**	6.3 ± 0.6 **b**	UA **d**	28.7 ± 0.2 **g**
ABR6	Necrosed leaf/“Cripps Pink” apple tree	Benton City, WA	*Priestia* sp.	3.2 ± 0.3 **c**	9.3 ± 0.6 **a**	5.8 ± 0.3 **c**	44.6 ± 2.0 **bc**
ABR8	Rootstock canker/M9	Sunnyside, WA	*Curtobacterium* sp.	1.7 ± 0.6 **d**	2.3 ± 0.6 **cd**	UA **d**	45.9 ± 0.8 **bc**
2175	Necrosed leaf/“Gala” apple tree	Kennewick, WA	*Bacillus* sp.	3.8 ± 0.3 **bc**	7.5 ± 1.5 **b**	11.0 ± 1.0 **a**	41.0 ± 1.2 **de**
BWC	Necrosed shoot/“Gala” apple tree	Kennewick, WA	*Bacillus* sp.	11.0 ± 1.0 **a**	3.7 ± 0.6 **c**	10.0 ± 0.0 **ab**	56.0 ± 1.4 **a**
Gram-negative							
EL51	Necrosed fruit/“Gala” apple tree	Kennewick, WA	*Rahnella* sp.	UA **e**	1.7 ± 0.6 **de**	UA **d**	36.1 ± 0.4 **f**
2186	Necrosed shoot/“Gala” apple tree	Kennewick, WA	*Erwinia* sp.	4.7 ± 0.6 **b**	UA **e**	UA **d**	47.5 ± 1.0 **b**
ABR1b	Symptomatic apple tree	WA	*Pantoea* sp.	UA **e**	UA **e**	UA **d**	40.6 ± 2.1 **e**
ABR1s	Symptomatic apple tree	WA	*Pantoea* sp.	UA **e**	UA **e**	UA **d**	28.6 ± 0.6 **g**
ABR5	Necrosed leaf/“Gala” apple tree	Kennewick, WA	*Pseudomonas* sp.	UA **e**	10.0 ± 0.5 **a**	UA **d**	44.2 ± 2.0 **bcd**
2180	Necrosed leaf/“Jazz” apple tree	Kennewick, WA	*Pseudomonas* sp.	UA **e**	9.7 ± 0.6 **a**	9.0 ± 2.7 **ab**	46.1 ± 0.9 **bc**
Yeast							
2176	Necrosed leaf/“Gala” apple tree	Kennewick, WA	*Rhodotorula* sp.	UA **e**	UA **e**	10.00 ± 1.0 **ab**	26.9 ± 0.4 **g**

^a^Identifications based on BLASTn analysis against NCBI refseq_rna database. For some isolates, high alignment scores among congeneric species were observed. For consistency, identifications are presented at the genus level.

^b^Amylase, protease and lipase activities quantified as halo diameter minus colony diameter. Siderophore production is quantified as percentage of Siderophore Units (% SU) by the liquid CAS assay. Data represents mean ± standard deviation (SD) (*n* = 3). UA, undetected (0 mm).

^c^In each column, different letters indicate statistically significant differences between the isolates (α = 0.05).

Antagonistic activity of these 13 isolates was tested against *E. amylovora* strain Z2274, also isolated from a symptomatic apple fruitlet in a Washington State orchard in 2022. Other *E. amylovora* isolates Z2304, Z2111, Z2129 and Z2143 were additionally used to confirm their biocontrol efficacy.

Unless otherwise specified, microorganism isolates were routinely cultured in Lysogeny Broth (LB) or agar (LA) plates. Fresh cultures were streaked on LA from −80°C glycerol stocks and incubated at 28°C for up to 72 h. Liquid cultures were initiated from single colonies and incubated overnight at 28°C with shaking (200 rpm). When required, overnight cultures were washed with phosphate buffered saline (PBS) and adjusted spectrophotometrically to an OD_600_ of 1 (10^9^ CFU/mL). For *Bacillus* spp. 2175 and BWC, *Priestia* sp. ABR6, and *Rhodotorula* sp. 2176, the same absorbance values led to cell suspensions at around 10^8^ CFU/mL and 10^7^ CFU/mL, respectively.

### 2.2 Detection of hydrolytic exoenzymes and siderophore production

Exoenzymatic activities were detected on solid media, as transparent halos (amylase, protease) or opaque precipitates (lipase) around colonies. Briefly, isolates were adjusted to 10^9^ CFU/mL in PBS and 2 μL of the cell suspension were placed on the agar surface in triplicate and allowed to dry. Plates were then incubated at 28°C for 48 h (amylase, protease) or 72 h (lipase). Amylase activity was detected on nutrient agar (Ward’s Science, Mississauga, ON) supplemented with 1% (w/v) soluble starch (Himedia, Mumbai, India), after flooding plates with 1% povidone-iodine solution. Protease activity was assessed on skim milk agar (per liter, 120 mL skim milk, 8 g tryptone, 2.5 g yeast extract, 1 g glucose, 15 g agar). Lipase activity was determined on nutrient agar (Ward’s Science, Mississauga, ON) amended with 0.5% (v/v) Tween-20 and 0.01% (w/v) CaCl_2_. For quantification, colony diameters were subtracted from exoenzymatic activity halo diameters.

Siderophore secretion was quantified using the chrome azurol S (CAS) liquid assay (Payne, 1994). Briefly, overnight LB cultures were rinsed with sterile distilled water and adjusted to 10^7^ CFU/mL in modified minimal MM9 medium (per L, 0.3 g KH_2_PO_4_, 0.5 g NaCl, 1 g NH_4_Cl, 0.3 g casein hydrolysate, 2 g glucose, 100 mM Tris●HCl, pH 7.4). Casein hydrolysate (Sigma-Aldrich, St. Louis, MO) was supplemented to support growth of isolates unable to grow in standard MM9. For each isolate, triplicate cultures were incubated for 48 h at 28°C with shaking (200 rpm). Supernatants were then mixed 1:1 with liquid CAS assay solution, prepared according to Payne (1994), and siderophore activity was measured at A_630_ nm after 1 h incubation at 25°C in the dark. Uninoculated MM9 medium was used as a blank, and a 1:1 mixture of uninoculated MM9 plus CAS reagent was used as a reference (r). The percentage of siderophore units (%SU) in each sample (s) was defined as [(A_630_(r) − A_630_(s)]/A_630_(r)] × 100 (Payne, 1994).

### 2.3 Detection of antagonistic activity by optimization of agar plug diffusion assay

An agar plug diffusion method (Mazza, 1983) was used to determine antagonistic activity against *E. amylovora*. Briefly, each BCA candidate was grown on the surface of LA plates for up to 5 days to allow metabolite diffusion into the agar. A 5- or 10-mm agar plug from the antagonist plates were excised using a sterile cork borer and transferred to fresh LA plates spread with 0.1 mL of *E. amylovora* Z2274 at 10^6^ CFU/mL in PBS. Plates were evaluated for growth inhibition around the plug after a 3-days incubation at 22°C or 28°C.

To optimize detection, we tested alternative media to LA, including King’s B agar (KBA) (King et al., 1954), LA supplemented with 0.4% glucose, 0.2 × diluted LA (with or without glucose), minimal medium AB (per L, 3 g K_2_HPO_4_, 1 g NaH_2_PO_4_, 1 g NH_4_Cl, 3 g MgSO_4_ 7H_2_O, 0.15 g KCl, 0.01 g CaCl_2_, 2.5 mg FeSO_4_ 7H_2_O) (Chilton et al., 1974), AB amended with 0.5% yeast extract, and M9 (per L, 6 g Na_2_HPO_4_, 3 g KH_2_PO_4_, 0.5 g NaCl, 1 g NH_4_Cl, 2 mM MgSO4) (Maniatis et al., 1982). Unless stated otherwise, AB and M9 minimal media were supplemented with 0.4% glucose as carbon source, and 0.05% nicotinic acid to allow *E. amylovora* growth (Starr and Mandel, 1950). Minimal media agar plates were prepared by autoclaving agar and salts separately and mixing them after media cooled down to around 50°C. Sugars were filter-sterilized and added before pouring plates. Media were prepared 24 h before the assay, poured and kept at room temperature until use. All assays were carried out in triplicate and repeated, at least, in two independent experiments.

### 2.4 Impact of carbon sources on *Erwinia amylovora* growth inhibition

Isolates were grown on LA or minimal AB medium supplemented with 0.4% glucose (ABGlc), sucrose (ABSuc), sorbitol (ABSor), fructose (ABFru) or glycerol (ABGly). The first four carbon sources represent the predominant sugars in immature fruit of *E. amylovora* hosts (Gu et al., 2021; Larson et al., 2023; Pleyerová et al., 2022; Yin et al., 2024; Watari et al., 2004) and are also present in different ratios in different hosts and tissues (Braun and Hildebrand, 2005; Choi et al., 2022; Filip et al., 2016; Tóth et al., 2003; Wallaart, 1980). Glycerol, while not a primary plant sugar, was included due to its role as a microbial carbon source and its potential to enhance antagonistic activity in certain bacteria (Prashanthi et al., 2021).

Agar plugs from plates containing 5-days-old BCA cultures were transferred to AB plates containing glucose, fructose, sorbitol, sucrose or glycerol, freshly spread with 0.1 ml of an *E. amylovora* Z2274 suspension at 10^6^ CFU/mL. After 3 days of incubation, growth inhibition halos were measured. The assay was performed in triplicate and repeated twice.

Throughout the manuscript, the notation LA → AB was used when plugs from BCA-seeded LA plates were transferred to *E. amylovora* seeded AB plates. The notation AB→AB was used when both BCA growth medium and the final transfer medium were AB agar.

### 2.5 Physical parameters influencing *in vitro* antagonistic activity against *E. amylovora*

*Erwinia amylovora* BCAs consistently producing clear growth inhibition halos were spread-plated on LB (*Bacillus* spp. isolates 2175 and BWC) or AB agar supplemented with 0.4% glucose (*Pseudomonas* spp. isolates 2180 and ABR5, *Rahnella* sp. EL51, *Priestia* sp. ABR6) and grown for 5 days at room temperature (22°C). Glucose was selected as the standard carbon source based on consistent production of well-defined inhibition zones on minimal medium. Agar plugs from these plates were transferred to AB agar plates freshly inoculated with *E. amylovora* Z2274 (10^6^ CFU/mL). For control conditions, plates were then incubated for 3 days at 22°C, and growth inhibition halo diameters were recorded after 72 h incubation. Different temperatures (22°C versus 28°C), pH (pH 7.0 versus pH 6.0 and pH 8.0) or glucose concentrations (0.4% versus 0.2% and 0.8% w/v) were tested in triplicate in parallel with all the strains.

### 2.6 Evaluation of biocontrol efficacy in immature fruitlets

Immature pears (*Pyrus communis* cv. Bartlett; approximately 2.5 cm × 4 cm) were harvested from a commercial orchard in Sunnyside (WA, USA) and stored at 4°C in the dark until use. Prior to inoculation, fruitlets were prepared by cutting peduncles at the base with pruners, washed with detergent, thoroughly rinsed with tap water and surface-disinfected by 60-s-immersion in 70% ethanol containing 0.5 mg/mL food-grade natamycin (MarkNature, CA, U.S.A.), followed by air-drying in a laminar flow hood.

The potential of BCAs to suppress fire blight symptoms was initially tested in detached immature pears cv. Bartlett. For co-inoculation assays, each fruitlet was wounded using a sterile 200 μL-pipette tip and 20 μL of a mixed suspension containing both *E. amylovora* and one BCA candidate (final concentration, 10^6^ CFU/wound per strain) was added to the wounding site. For preventive treatments, BCA candidate inoculation (10^6^ CFU/wound) was done 24 h before *E. amylovora* inoculation (10^6^ CFU/wound). Positive controls consisted of wounded fruit inoculated with *E. amylovora* alone (co-inoculation treatments) and fruit wounded and inoculated with 10 μL PBS 24 h before *E. amylovora* inoculation (preventive treatments). Negative controls in both cases were wounded fruit treated with PBS. Additional controls consisted of fruit inoculated with BCAs alone, to assess potential pathogenicity. Unless stated differently, the term “days post-inoculation (dpi)” refers to the number of days after *E. amylovora* (not BCA) inoculation.

Inoculated fruitlets (*n* = 6–7 per treatment) were placed in petri dishes within sterile aluminum trays lined with wet paper towels and tightly covered with aluminum foil. Trays were incubated at 25°C for 6 days after *E. amylovora* inoculation. Disease severity was rated using a severity index scale from 0 to 4, where 0 indicated no symptoms; 1, necrosis restricted to the inoculation site; 2, necrosis extending up to 20% of half-fruit surface; 3, necrosis covering 21%–40% of half-fruit surface; and 4, necrosis affecting more than 40% of half-fruit surface. Biocontrol capacity was assessed in relation to the average severity index and percentage of asymptomatic fruit per treatment, as compared to the corresponding controls inoculated with *E. amylovora*.

### 2.7 Assessment of interactions among BCAs and capacity of pairwise combinations for fire blight suppression

Biocontrol efficacy and effect of interactions between BCAs in disease suppression were assessed using detached immature apples (*Malus domestica* cv. Pink Lady; approximately 3 cm × 4 cm) and a preventive treatment regime as described above. Apples were harvested from WSU-Roza Farm in Prosser (WA, USA), washed with soap and disinfected as described for pears with 0.5 mg/mL natamycin in 70% ethanol.

Biocontrol agents were applied to fruit wounds (10^6^ CFU/wound) either alone or in all possible pairwise combinations with the remaining BCAs (*n* = 5 fruit per treatment and a total of 78 BCA combinations plus 13 single-BCA treatments). *E. amylovora* was inoculated 24 h later. Fruits were incubated for 2 weeks at 25°C in moist chambers. Controls consisted of wounded fruitlets treated with sterile PBS followed by *E. amylovora* inoculation 24 h later. Negative controls were inoculated with PBS alone. Symptom progression was monitored over time, with assessments recorded at 9 and 14 dpi. Disease severity was rated on a 0–6 scale, where 0 indicated no symptoms; 1, necrosis confined to the inoculation site; 2, necrosis extending up to 20% of the half-fruit surface; 3, necrosis covering 21%–40% of half-fruit surface; 4 necrosis occupying 41%–100% of half-fruit surface; 5, necrosis extending beyond half fruit, with green patches still observable; 6, complete necrosis of the entire fruit.

Biocontrol efficacy of individual BCAs was assessed using severity index values and percentage of asymptomatic fruits. To assess net biocontrol efficacy of pairwise BCA combinations, we used two different parameters, the percentage disease severity reduction (%DSR), linked to disease severity, and the percentage of control efficacy (%CE), linked to disease incidence. %DSR was determined as %DSR = 100 × (SI*_*Ea*_* − SI*_*t*_*)/SI*_*Ea*_*, where SI*_*Ea*_* represents the mean severity index in *E. amylovora*-inoculated control fruit and SI*_*t*_* is the mean severity index of treated fruit. %CE was calculated as %CE = 100 × (SF*_*Ea*_* − SF*_*t*_*)/(SF*_*Ea*_*), where SF*_*Ea*_* is the number of symptomatic fruits in *E. amylovora*-treated controls and NS*_*t*_* is the number of symptomatic fruits following single or paired BCA treatment.

Interactions between paired BCAs were analyzed to classify their combined effect on fire blight suppression as synergistic, antagonistic or additive/neutral. For both %DSR and %CE, the net interaction value (Δ) was defined as the difference between the observed and expected values: Δ %DSR = Observed %DSR − Expected %DSR, and Δ %CE = Observed %CE − Expected %CE. Expected %DSR and %CE values were calculated using two different frameworks: Bliss independence with overlap and the “best single agent,” also known as “highest single agent.” Bliss independence calculates the expected effect as if the two BCAs acted independently without interfering with each other, Expected %DSR = DSR_1_ + DSR_2_ − (DSR_1_ × DSR_2_) and Expected %CE = CE_1_ + CE_2_ − (CE_1_ × CE_2_), with DSR_1_, CE_1_ and DSR_2_ and CE_2_ representing the efficacy of each strain applied individually (Bliss, 1956; Xu et al., 2011; Xu and Jeger, 2013a). The “best single agent” model is more conservative and sets the expected effect to that of the best-performing BCA in the pair, Expected %DSR = Max (%DSR_1_, %DSR_2_) and Expected %CE = Max (%CE_1_, %CE_2_) (Xu and Jeger, 2013b).

To classify interactions, we applied a pre-defined threshold of 5%, and only deviations exceeding this margin were considered different from neutral. Synergistic effects in disease suppression were assigned to Δ %DSR and Δ %CE values equal or over 5%; antagonistic effects, to Δ values equal or below −5%; and neutral/additive effects to Δ values between −5% and +5%. Synergistic and antagonistic effects were further classified as moderate when 5% ≤ | Δ— < 20%, or high when | Δ— ≥ 20%. For each isolate, its behavioral trend toward synergism or antagonism was evaluated based on the median net interaction values across partnerships as well as the distribution of its interaction categories.

### 2.8 *E. amylovora* and BCA population dynamics in immature “Pink Lady” apples

A paired treatment with *Erwinia* sp. 2186 and *Rahnella* sp. EL51 was chosen based on best absolute %DSR and %CE data from the experiments above. Given that *E. amylovora*, *Erwinia* sp. 2186 and *Rahnella* sp. EL51 form similar colonies on LA plates, spontaneous mutant strains of *E. amylovora* Z2274 and *Rahnella* sp. EL51, resistant to rifampicin (Rif) and nalidixic acid (Nal), respectively (Z2274*^Rif^* and EL51*^Nal^*), were used for inoculations, and plate counts were carried out on LA with 100 μg/mL Rif for Z2274*^Rif^*, and 100 μg/mL Nal for EL51*^Nal^*. LA amended with 12.5 μg/mL oxytetracycline (OTc) was used for counting *Erwinia* sp. 2186, which was naturally resistant to this antibiotic.

Apple fruitlets were disinfected as described above and inoculated following a preventive treatment regime. Treatments included individual applications of *Rahnella* sp. EL51, *Erwinia* sp. 2186, and *E. amylovora* Z2274, as well as all combinations of two and three strains (10^6^ CFU per strain and fruit). Each treatment was assayed with 3 replicate fruit per time point, at 0, 3, 10 and 15 dpi. At each time point, 3 fruitlets were weighted before processing and homogenized with a hammer in a zip-log bag with 40 mL sterile PBS. After serial dilutions, plate counts of *E. amylovora* Z2274*^Rif^*, *Rahnella* sp. EL51*^Nal^* and *Erwinia* sp. 2186 were performed on LA + Rif, LA + Nal and LA + OTc, respectively. After 24–48 h incubation at 28°C, plate counts and each fruit’s weight were used to normalize data to Log CFU/g fruit.

### 2.9 Effect of BCA growth on media pH

Overnight cultures of *Erwinia* sp. 2186, *Rahnella* sp. EL51 and *E. amylovora* Z2274 rinsed twice with PBS were inoculated in fresh medium at a starting A_600_ nm of 0.001 (10^6^ CFU/mL) alone and in combinations of two and three strains. Cultures were performed in parallel in LB and in minimal medium AB with sugar content mimicking that of unripe “Gala” apples, AB components plus a final carbohydrate concentration arbitrarily set to 0.4% w/v, composed of 36.1% fructose, 31.1% Glucose and 30.8% Sorbitol and 2.0% starch (Larson et al., 2023). After overnight growth at 28°C with shaking (180 rpm), cells were pelleted by centrifugation, supernatants filter-sterilized and pH measured with a Mettler Toledo pH-meter (model F20-Standard FiveEasy). The assay was performed in triplicate.

### 2.10 Statistical analysis

All data were analyzed in GraphPad Prism v10.5 using standard parametric or non-parametric procedures matched to the distribution of residuals. Single-factor experiments (effects of pH, carbon source or temperature on inhibition halos) were evaluated with one-way ANOVA or Kruskal-Wallis tests (when homoscedasticity and normality assumptions were not met), followed by Dunn’s *post-hoc* tests. In normally distributed data sets with significant heterogeneity of variances, Brown-Forsythe ANOVA with Dunnett’s T3 were employed to assess pairwise comparisons. Two-factor datasets (time × strain or medium × strain) were evaluated with two-way ANOVA. After ANOVA tests, significant interactions or main effects were further explored with Tukey’s tests for pairwise comparisons or Dunnett’s tests when each treatment was compared with the designated control. CFU counts in population dynamics data were log-transformed to stabilize variances. Numerical results were reported as means ± standard deviation (SD) from three or more replicates. Box-and-whisker plots were used to display medians, inter-quartile ranges and overall data ranges. The relationships between single-BCA and paired-BCA efficacies were assessed using Spearman’s rank correlation due to the non-normal distribution of %CE data and to ensure comparability between %DSR and %CE results. A significant threshold of α = 0.05 was adopted for all tests.

## 3 Results

### 3.1 Identity and phenotype of microbial isolates

A diverse set of microorganisms co-isolated with *E. amylovora* from symptomatic apple tissues of commercial orchards in WA State was selected for the study (Table 1). In most cases, partial 16s rRNA (bacteria) and 23S rRNA (yeast) gene sequencing results revealed high similarity scores among species of the same genus, so all isolates were identified at the genus level for consistency. Gram-positives belonged to Bacillaceae (*Bacillus* spp. 2175 and BWC, *Priestia* sp. ABR6) and Microbacteriaceae families (*Microbacterium* sp. 2173, *Microbacterium* sp. ABR10 and *Curtobacterium* sp. ABR8). Among the Gram-negatives, three isolates belonged to the Erwiniaceae family, including *Erwinia* sp. 2186, *Pantoea* sp. ABR1b and *Pantoea* sp. ABR1s; one isolate, *Rahnella* sp. EL51, to the Yersiniaceae family; and the remaining two isolates were pseudomonads within the *P. syringae* (*Pseudomonas* sp. ABR5) and *P. fluorescens* (*Pseudomonas* sp. 2180) species complexes. Regarding the yeast, it was identified as *Rhodotorula* sp. 2176, a pink-pigmented member of the Sporidiobolaceae family.

Except for *Microbacterium* sp. ABR10, Gram-positives were the main amylase producers, with *Bacillus* sp. BWC and *Curtobacterium* sp. ABR8 exhibiting the highest and lowest amylase activity (11.0 ± 1.0 mm and 1.7 ± 0.6 mm, respectively). Among the Gram-negatives, only *Erwinia* sp. 2186 showed some amylase activity (4.7 ± 0.6 mm) comparable to that of *Bacillus* sp. 2175 (3.8 ± 0.3 mm) (Table 1). In contrast, Gram-negatives, especially those of the genus *Pseudomonas*, exhibited high protease activity, with isolates ABR5, and 2180 producing halos between 9.7 and 10.0 mm. Gram-positives *Priestia* sp. ABR6, *Microbacterium* sp. ABR10, *Curtobacterium* sp. ABR8 and the *Bacillus* spp. 2175 and BWC also produced protease, with halo diameters ranging between 2.3 and 9.3 mm. The main lipase producers were the two *Bacillus* spp. 2175 (11.0 ± 1.0 mm) and BWC (10.0 ± 0.0 mm), and the yeast *Rhodotorula* sp. 2176 (10.0 ± 0.0 mm) followed by the *Pseudomonas* isolates 2180 (9.0 ± 2.7 mm) and *Priestia* sp. ABR6 (5.8 ± 0.3 mm) (Table 1). The CAS liquid assay indicated that supernatants from all the isolates (including the yeast *Rhodotorula* sp. 2176), contained siderophores, with the top producers being the *Bacillus* sp. BWC (55.9 ± 1.4% SUs), followed by *Erwinia* sp. 2186 (47.5 ± 1.0% SUs) and *Pseudomonas* sp. 2180 (46.1 ± 0.9% SUs) (Table 1).

### 3.2 Optimized detection of *in vitro* antagonistic activity

Optimization of an agar plug assay revealed that *in vitro* antagonism against *E. amylovora* was strongly influenced by culture media. On complex KBA medium, most isolates induced changes in *E. amylovora*’s growth and mucoidy, rather than producing clear inhibition halos (Supplementary Figure 1). Testing on LA, 0.2xLA, and their glucose-supplemented variants also rarely improved halo detection. In contrast, minimal media like AB and M9 enhanced the detection of growth inhibition zones of three main types: (i) sharp and defined inhibition zones, produced by *Priestia* sp. ABR6, *Bacillus* spp. 2175 and BWC, *Rahnella* sp. EL51 and *Pseudomonas* spp. ABR5 and 2180; (ii) inhibition zones with defined margins, where *E. amylovora* grew but at lower density (associated with *Erwinia* sp. 2186); (iii) subtle inhibition zones without defined borders, typically developed by *Microbacterium* spp. 2173 and ABR10, and *Rhodotorula* sp. 2176. Amendment of minimal media with yeast extract, neutralized formation of inhibition zones around plugs (Supplementary Figure 1). Assay conditions involving pre-growth of Gram-negatives and *Priestia* sp. ABR6 on AB agar and of *Bacillus* spp. 2175 and BWC on LA before transfer to *E. amylovora*-seeded AB agar, enabled optimal detection of antagonistic activity against multiple *E. amylovora* strains, with slight, strain-dependent variations in efficacy (Supplementary Figure 2).

### 3.3 Carbon-source-driven variation in antagonistic activity

**FIGURE 1:**
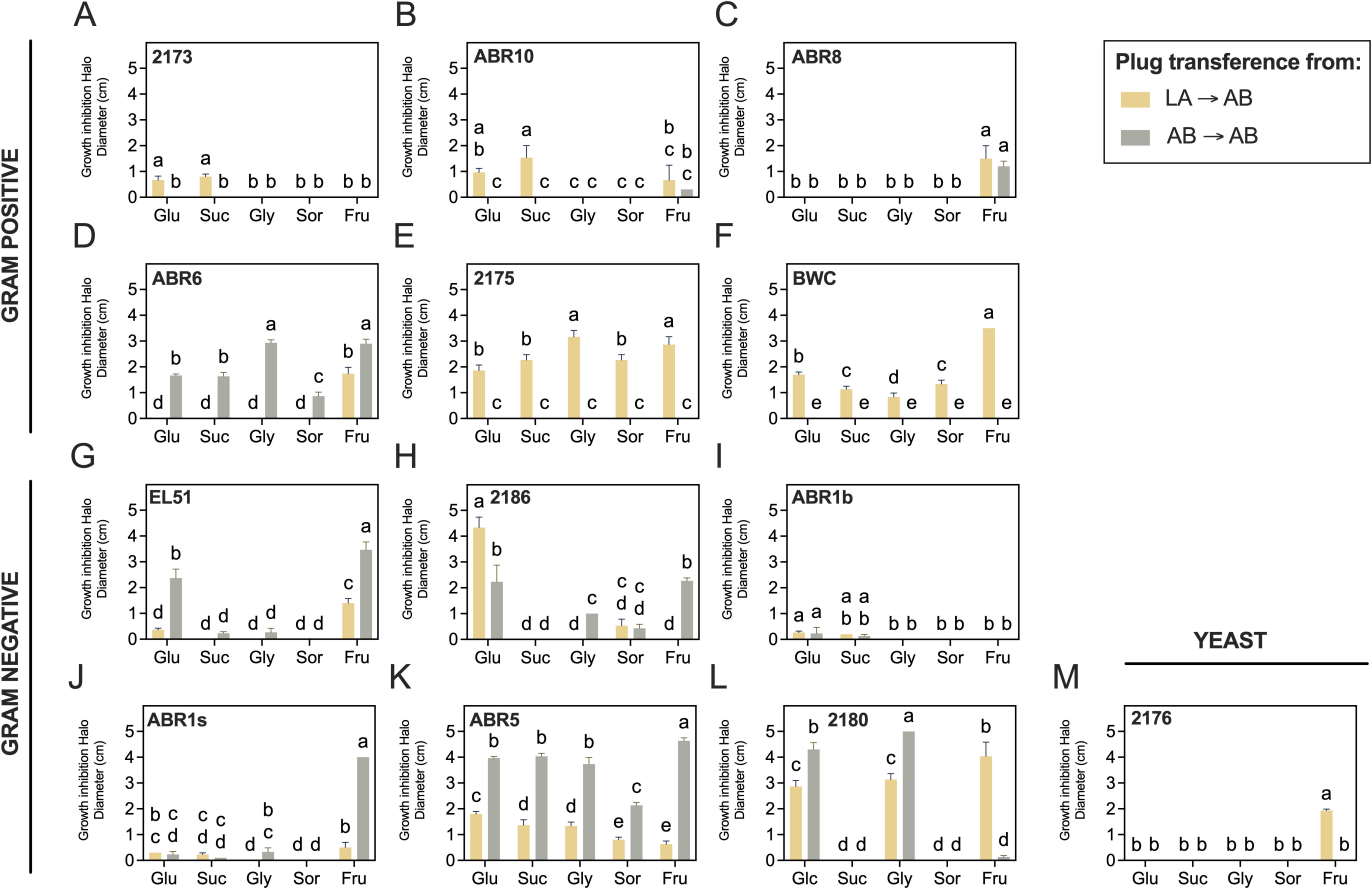
Effect of carbon source in *in vitro* antagonistic activity of bacterial and yeast microorganisms against *E. amylovora* by an agar plug assay. Antagonist plugs (10 mm diameter) were cut from 5-days-old cultures grown at room temperature (22°C) either on LA (yellow) or minimal AB agar, both supplemented with either 0.4% glucose (Glu), sucrose (Suc), glycerol (Gly), sorbitol (Sor) or fructose (Fru) (gray) and transferred to AB agar plates containing the same carbon source and freshly seeded with *E. amylovora*. Each graph shows the antagonist activity of one strain, and bars represent mean values ± standard deviation of three plates per strain and condition. Different letters indicate significant differences between the compared groups by Tukey’s tests (α = 0.05). **(A)** *Microbacterium* sp. 2173; **(B)** *Microbacterium* sp. ABR10; **(C)** *Curtobacterium* sp. ABR8; **(D)** *Priestia* sp. ABR6; **(E)** *Bacillus* sp. 2175; **(F)** *Bacillus* sp. BWC; **(G)** *Rahnella* sp. EL51; **(H)** *Erwinia* sp. 2186; **(I)** *Pantoea* sp. ABR1b; **(J)** *Pantoea* sp. ABR1s; **(K)** *Pseudomonas* sp. ABR5; **(L)** *Pseudomonas* sp. 2180; **(M)** *Rhodotorula* sp. 2176.

The most effective plug transfer conditions (LA→AB and AB→AB) were used to assess the impact of carbon sources on inhibition zone development (Figure 1). Results indicated that carbon sources had the most significant effect in halo development (*P* < 0.0001), accounting for an average of 47.6% (±26.2) of the observed variability. The plug transfer strategy used for halo detection (LA→AB vs. AB→AB) was also significant for most strains (*P* ≤ 0.0362), contributing an average of 37.5% (±31.8) to the observed variability. The interaction between carbon source and the plug transfer strategy was also significant for most isolates (*P* < 0.0001) (Figure 1), confirming that the effect of each factor was strain dependent.

Sorbitol typically produced the smallest zones, followed by sucrose and glycerol. In contrast, fructose and glucose yielded the largest inhibition zones for most strains (Figure 1). Most Gram-positive isolates developed larger inhibition zones when pre-cultured on rich LA and transferred to AB (LA AB), irrespective of the carbon source (Figures 1A,B,E,F). In contrast, *Priestia* sp. ABR6 developed better activity when grown on minimal AB, regardless of the carbon source (Figure 1D). Isolates of the Bacillaceae family (ABR6, 2175 and BWC) showed the strongest antagonistic activities among the Gram-positives, producing defined inhibition zones with no residual pathogen growth. *Priestia* sp. ABR6 and *Bacillus* sp. 2175 were the most effective on glycerol- or fructose-amended AB, achieving maximum inhibition zones of approximately 2.9 and 3.8 cm, respectively (Figures 1D,E). LA-grown *Bacillus* sp. BWC showed peak activity on AB plus fructose, with inhibition zones of 3.5 cm (Figure 1F). The lowest *in vitro* activity among Gram-positives was observed in isolates of the Microbacteriaceae family (2173, ABR8, ABR10). The largest inhibition zones in these cases were detected on glucose-, fructose- or sucrose-amended AB, with inhibition zones never surpassing 2.5 cm diameter (Figures 1A–C).

Most Gram-negative isolates showed enhanced activity when grown on and transferred to AB (AB AB) (Figures 1G–L). An exception was *Erwinia* sp. 2186, which developed larger inhibition zones when grown on LA and transferred to glucose-amended AB (LA ABGlu) (Figures 1H,L). Among Gram-negatives, only *Rahnella* sp. EL51 and *Pseudomonas* spp. ABR5 and 2180 produced well-defined inhibition zones with complete growth inhibition. Others, like *Erwinia* sp. 2186, produced diffuse halos with defined margins, allowing limited *E. amylovora* growth. AB-grown *Rahnella* sp. EL51 showed the highest activity on AB with fructose (3.5 ± 0.3 cm diameter), followed by glucose (2.4 ± 0.4 cm) but minimal activity on sucrose- and glycerol-amended AB (around 0.3 cm) (Figure 1G). *Pseudomonas* sp. ABR5 showed high activity on all carbon sources, with the largest inhibition zones on AB plus fructose (4.6 ± 0.1 cm), followed by glucose, sucrose and glycerol (all around 3.9 cm), and sorbitol (2.1 ± 0.1 cm) (Figure 1K). Both *Pseudomonas* spp. 2180 and ABR5 showed activity when grown on LA and transferred to AB (LA AB), though producing smaller zones (Figures 1K,L). Finally, the fluorescent pseudomonad 2180 developed the largest inhibition zones when grown and transferred to AB containing glycerol (5.0 ± 0.0 cm) or glucose (4.3 ± 0.3 cm), but activity was strongly reduced on AB with fructose (0.1 ± 0.1 cm) and absent on AB with sorbitol (Figure 1L). Interestingly, the activity on fructose-amended AB was high (4.0 ± 0.6 cm) when this strain was grown on LA, but nearly absent when grown on AB (Figure 1L).

The yeast *Rhodotorula* sp. 2176 only showed significant activity when grown on LA and transferred to fructose-amended AB (LA ABFru), producing inhibition zones of 2.9 ± 0.1 cm (Figure 1M). None of the remaining tested conditions led to detectable inhibition zones.

### 3.4 Effect of pH, incubation temperature and glucose concentration on growth inhibition zone development

**FIGURE 2:**
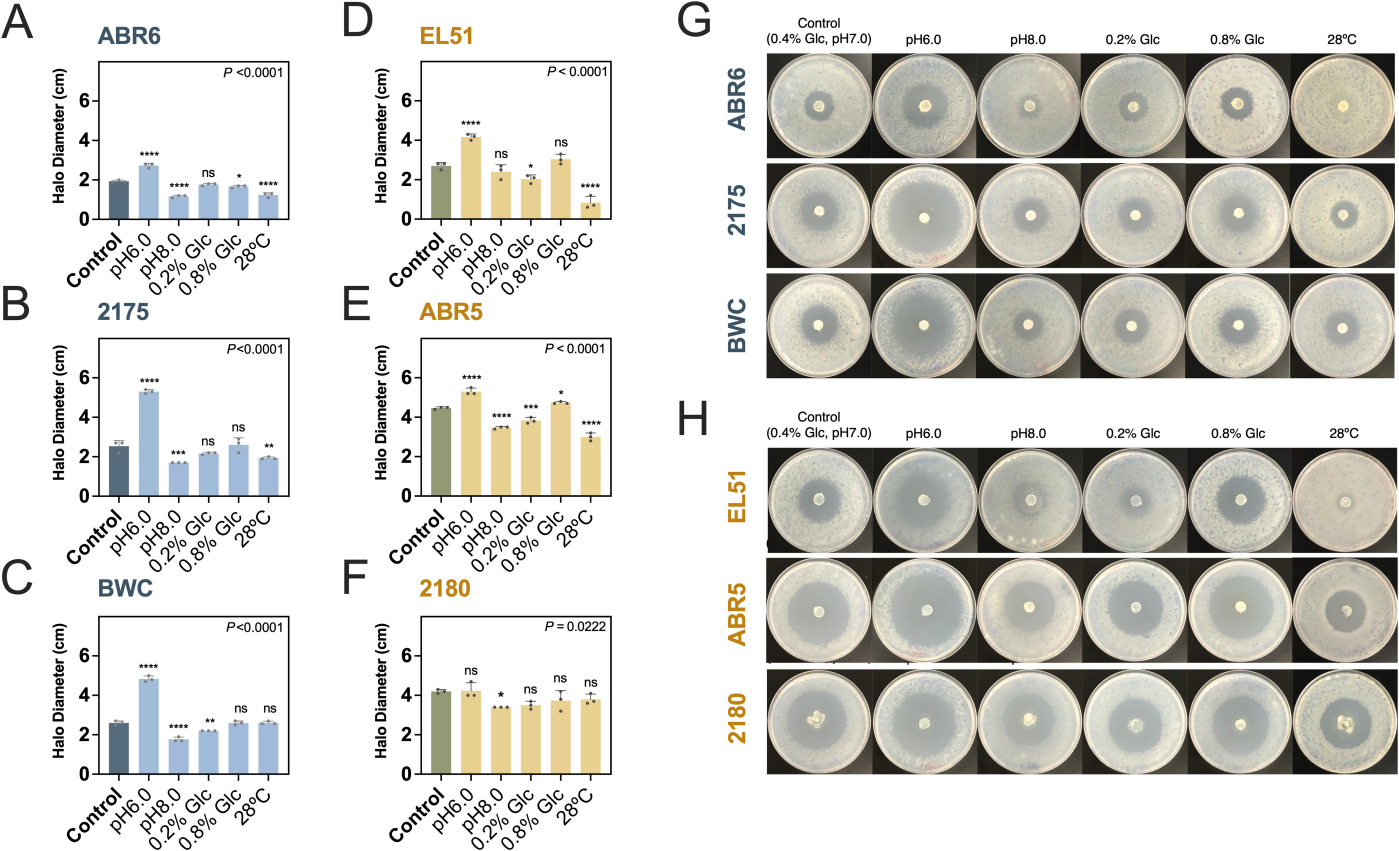
Effect of pH, glucose concentration and temperature on *in vitro* antagonistic activity of Gram-positive and Gram-negative antagonists against *E. amylovora*. Control conditions consisted of growth on AB with 0.4% glucose, pH 7.0 at room temperature (22°C). Variations of pH, glucose concentration and incubation temperature were explored in representative **(A–C)** Gram-positive (blue) and **(D–F)** Gram-negative (yellow) isolates. Asterisks denote statistically significant differences between the indicated variable and control conditions: *P < 0.05; **P < 0.01; ***P < 0.001; ****P < 0.0001; ns, non-significant (*P* > 0.05). **(G,H)** Representative images of the assay with Gram-positives **(G)** and Gram-negatives **(H)**. *Priestia* sp. ABR6, *Bacillus* sp. 2175, *Bacillus* sp. BWC, *Rahnella* sp. EL51, *Pseudomonas* sp. ABR5, *Pseudomonas* sp. 2180.

The effects of pH, glucose concentrations and incubation temperature on *E. amylovora* growth inhibition were analyzed using representative Gram-positive and Gram-negative isolates that produced strong, well-defined inhibition zones (Figure 2).

All three Gram-positive isolates exhibited significant variations in antagonistic activity in response to changes in pH (*P* < 0.001). *Priestia* sp. ABR6, and the two *Bacillus* sp. isolates 2175 and BWC showed 1. 4-, 2.1- and 1.9-fold increased inhibition zones at pH6.0 (*P* < 0.0001), respectively, with respect to control conditions (Figures 2A–C,G). In contrast, *Bacillus* sp. isolates 2175 and BWC showed significantly reduced inhibition zones at pH 8.0 (around 1.7 cm) compared to control conditions (2.6 cm) (*P* < 0.001) (Figures 2B,C,G). Temperature also affected the development of inhibition zones. Two out of the three Gram-positives (*Priestia* sp. ABR6 and *Bacillus* sp. 2175) exhibited significantly reduced inhibition zones when incubated at 28°C as compared to 22°C (Figures 2A,B,G). No significant effect was observed for *Bacillus* sp. BWC (*P* > 0.9999) (Figures 2C,G).

Among the Gram-negative isolates, pH, carbon concentration and incubation temperature had more pronounced effects on *Rahnella* sp. EL51 (*P* < 0.0001) (Figures 2D,H) and *Pseudomonas* sp. ABR5 (*P* < 0.0001) (Figures 2E,H) than on *Pseudomonas* sp. 2180 (*P* = 0.0222) (Figures 2F,H). For *Rahnella* sp. EL51 and *Pseudomonas* sp. ABR5, inhibition zones increased 1.5 and 1.2-fold at pH6.0 as compared to pH 7.0, respectively, but no effects were observed in *Pseudomonas* sp. 2180 (*P* = 0.8937) (Figures 2D–F,H). In contrast, pH (8.0) negatively affected the antagonistic activities of both *Pseudomonas* sp. ABR5 and 2180 (*P* ≤ 0.0327), but not of *Rahnella* sp. EL51 (*P* = 0.2549).

Reducing glucose concentration only impaired antagonistic activities in *Rahnella* sp. EL51 and *Pseudomonas* sp. ABR5, which decreased inhibition halo sizes to 2.0 ± 0.2 cm (*P* = 0.0232) and 3.8 ± 0.2 cm (*P* < 0.0001), respectively (Figures 2D,E). Increasing glucose to 0.8% slightly improved antagonistic activities in both isolates, though this was only significant in ABR5 (*P* = 0.0285). In contrast, higher incubation temperature (28°C) drastically reduced *E. amylovora* growth inhibition by EL51 and ABR5, dropping inhibition zones by approximately 3.3-fold and 1.5-fold, respectively (*P* < 0.0001) (Figures 2D,E,H). Neither glucose concentration nor temperature significantly affected antagonistic activity of *Pseudomonas* sp. 2180 (*P* ≥ 0.0555) (Figures 2F,H).

### 3.5 Antagonistic activity of BCAs against *E. amylovora* on immature pears and apples

**FIGURE 3:**
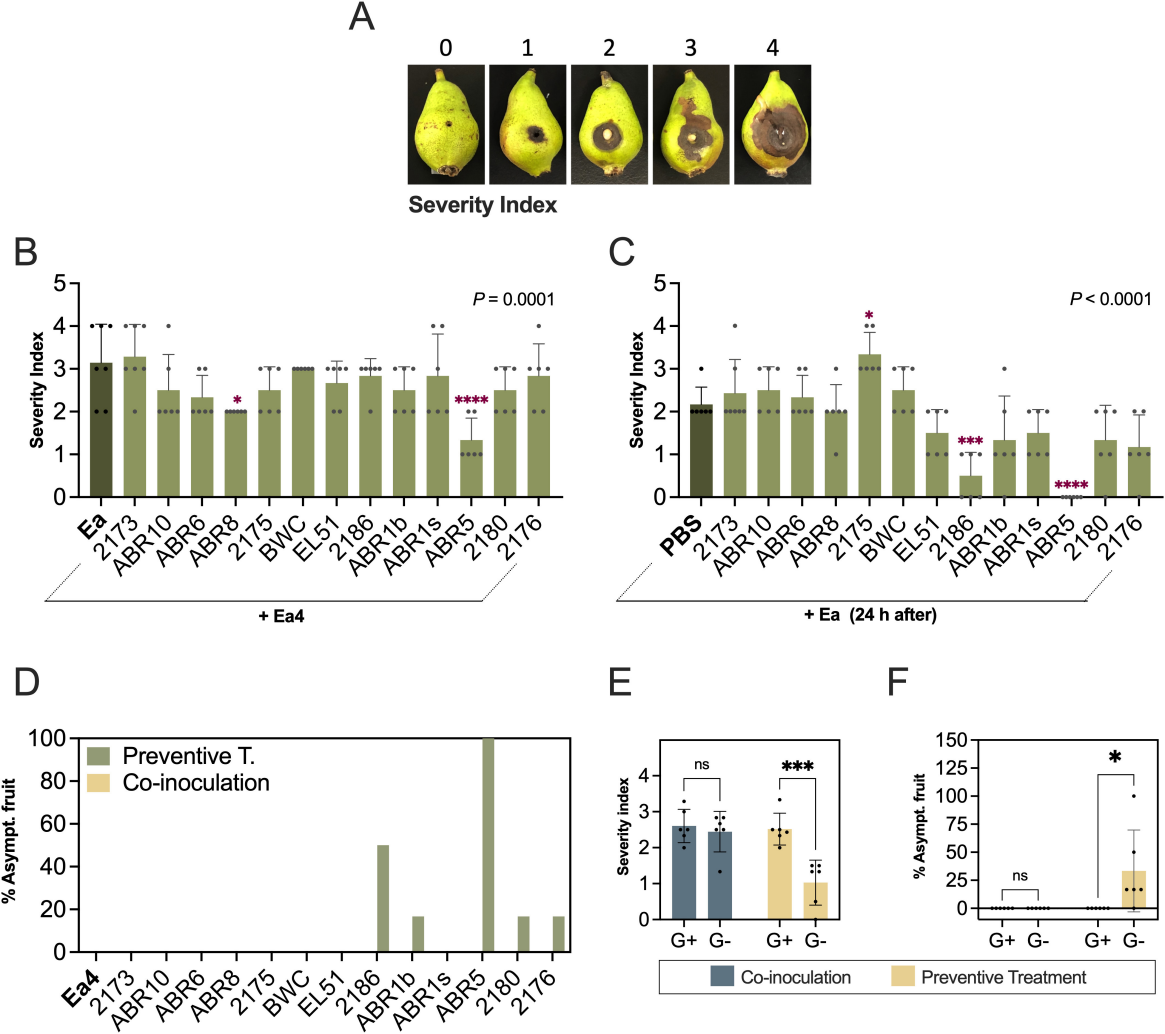
Antagonistic activity of bacterial and yeast isolates against *E. amylovora* in immature “Bartlett” pears. **(A)** Severity index scale used for rating fire blight symptoms in pear fruitlets. **(B)** Co-inoculation treatments. **(C)** Preventive treatments. Data represents mean values (±standard deviation) from 6 to 7 fruit per isolate. **(D)** Percentage of asymptomatic fruitlets at 6 dpi. **(E,F)** Effect of organism type (Gram-positives, G+; Gram-negatives, G–) in mean severity index values **(E)** and percentage of asymptomatic fruit **(F)**. Each dot is a mean value from 6 to 7 fruits. Asterisks show significant differences between treatments, or G+ and G– organisms: *P ≤ 0.05; ***P = 0.001; ****P ≤ 0.0001; ns, non-significant, *P* > 0.05. Gram-positive isolates: *Microbacterium* sp. 2173; *Microbacterium* sp. ABR10, *Priestia* sp. ABR6, *Curtobacterium* sp. ABR8; *Bacillus* sp. 2175, *Bacillus* sp. BWC; Gram-negatives: *Rahnella* sp. EL51, *Erwinia* sp. 2186; *Pantoea* sp. ABR1b, *Pantoea* sp. ABR1s, *Pseudomonas* sp. ABR5, *Pseudomonas* sp. 2180; yeast: *Rhodotorula* sp. 2176.

Two inoculation regimes on immature “Bartlett” pears, co-inoculation (simultaneous application of antagonist and pathogen) and preventive treatment (antagonist applied 24 h before *E. amylovora*) were used to evaluate biocontrol efficacy of BCAs. Disease suppression was determined based on a disease severity index (Figure 3A) and the number of asymptomatic fruits at 6 dpi.

In co-inoculation assays, most BCA treatments did not significantly reduce disease severity or the number of infected fruits compared to the *E. amylovora*-only control (mean SI 3.1 ± 0.9; Figures 3B,D, Supplementary Figure 3). Only two isolates significantly reduced symptom severity: *Curtobacterium* sp. ABR8 (mean SI: 2.0 ± 0.0; *P* = 0.0184) and *Pseudomonas* sp. ABR5 (mean SI: 1.3 ± 0.5; *P* < 0.0001) (Figure 3B). Interestingly, fruit inoculated with ABR5 alone developed small necrotic lesions distinct from typical fire blight symptoms (Supplementary Figure 3). Unlike characteristic tissue water-soaking, rapidly progressing necrosis and ooze accumulation caused by *E. amylovora*, ABR5-induced lesions were drier, darker, and remained localized near the inoculation site.

Preventive BCA applications yielded more inter-strain variability than co-inoculations (Figure 3B). Among all strains, two Gram-negative isolates (*Pseudomonas* sp. ABR5 and *Erwinia* sp. 2186) significatively reduced symptoms at 6 dpi. *Pseudomonas* sp. ABR5 reduced 100% fire blight symptoms, with replicate fruit showing no symptoms or only ABR5-associated symptoms at 6 dpi (Figures 3C,D, Supplementary Figure 3). *Erwinia* sp. 2186 also significantly reduced disease severity (mean SI: 0.5 ± 0.6; *P* = 0.0002) and the number of symptomatic fruits (50%) when applied preventively (Figures 3C,D, Supplementary Figure 3). *Pantoea* sp. ABR1b, *Pseudomonas* sp. 2180 and the yeast *Rhodotorula* sp. 2176 also decreased the number of infected fruits (Figure 3D), but with no overall effects on symptom severity (Figure 3C).

Overall, preventive treatments with Gram-negative BCAs provided better symptom suppression (mean SI: 1.0 ± 0.6) as compared to Gram-positive organisms (SI 2.5 ± 0.4) (*P* = 0.0002; Figure 3E) and increased the percentage of asymptomatic fruits to 33.4% as compared to 0% of treatments with Gram-positives (*P* = 0.0368; Figure 3F). In contrast, co-inoculation assays showed no significant differences in disease severity (*P* = 0.8474) or the percentage of asymptomatic fruits (*P* > 0.9999) associated with the bacteria type (Figures 3E,F).

**FIGURE 4:**
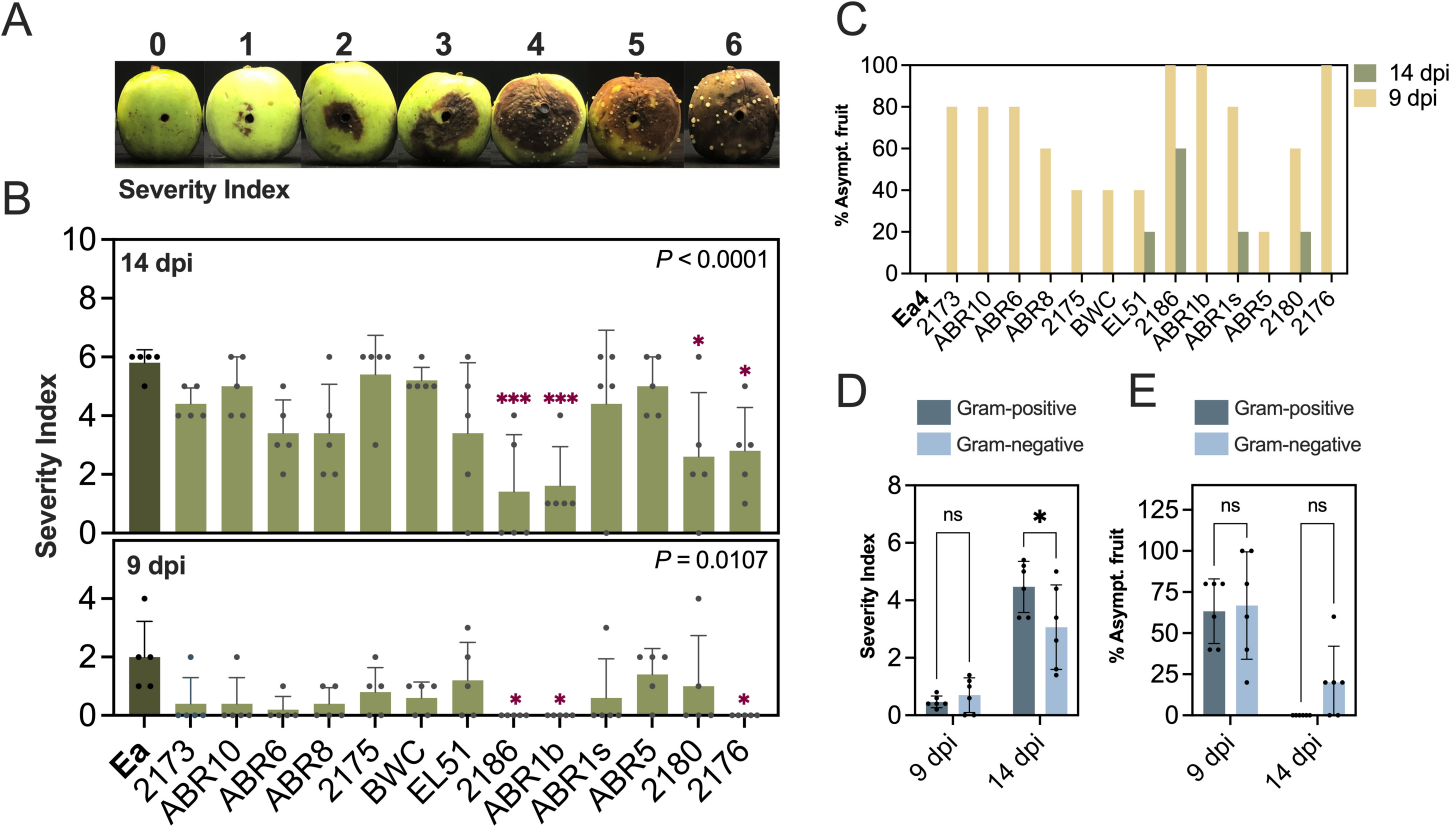
Antagonistic activity of bacterial and yeast isolates against *E. amylovora* in immature “Pink Lady” apples under pre-inoculation conditions. **(A)** Severity index scale used for symptom rating in apple fruitlets. **(B)** Mean severity index values (±standard deviation) from 5 fruit per isolate and **(C)** percent asymptomatic fruit at 9 and 14 dpi. **(D,E)** Effect of organism type (Gram-positives, G+; Gram-negatives, G–) in mean severity index **(D)** and percentage of asymptomatic fruit **(E)**. Each dot is a mean value from 5 fruit. Asterisks denote treatments significantly reducing disease severity **(B)** or differences between G+ and G– organisms (DE) at 9 and 14 dpi: *P < 0.05; ***P < 0.001. Gram-positive isolates: *Microbacterium* sp. 2173; *Microbacterium* sp. ABR10, *Priestia* sp. ABR6, *Curtobacterium* sp. ABR8; *Bacillus* sp. 2175, *Bacillus* sp. BWC; Gram-negatives: *Rahnella* sp. EL51, *Erwinia* sp. 2186; *Pantoea* sp. ABR1b, *Pantoea* sp. ABR1s, *Pseudomonas* sp. ABR5, *Pseudomonas* sp. 2180; yeast: *Rhodotorula* sp. 2176.

In a different set of experiments, we used immature “Pink Lady” apples to assess the efficacy of single BCA treatments in suppressing fire blight symptoms (Figure 4). Disease progression in control fruit inoculated only with *E. amylovora* was slower than in pears. Of the BCAs tested, *Erwinia* sp. 2186, *Pantoea* sp. ABR1b and *Rhodotorula* sp. 2176 completely suppressed symptoms at 9 dpi (*P* ≤ 0.0126) and significantly reduced SI at 14 dpi (*P* ≤ 0.0308) (Figure 4B). *Pseudomonas* sp. 2180 also significantly suppressed symptoms at 14 dpi (*P* = 0.0177) (Figure 4B). None of the BCAs were pathogenic on “Pink Lady” apples (data not shown).

Treatment of “Pink Lady” apples with all BCAs led to 20%–100% symptom suppression at 9 dpi (Figure 4C). By 14 dpi, only four BCAs (*Rahnella* sp. EL51, *Erwinia* sp. 2186, *Pantoea* sp. ABR1b and *Pseudomonas* sp. 2180) provided certain control, with 20% of fruits remaining asymptomatic in most cases and *Erwinia* sp. 2186 being the most effective BCA, with 60% of fruits remaining asymptomatic at the end of the experiment (Figure 4C).

Grouping antagonists by Gram-type revealed a trend similar to that observed in pears. At 14 dpi, fruit treated with Gram-negative bacteria had less severe infections (mean SI 3.1 ± 1.5) and a higher percentage of asymptomatic fruits (20.0% ± 22%) than those treated with Gram-positives (mean SI: 4.5 ± 0.9; 0.0% asymptomatic fruit). However, only the difference in mean SI between the two groups was statistically significant (*P* = 0.0307) (Figures 4D,E).

### 3.6 Pairwise BCA combinations enhanced fire blight suppression on immature apples

**FIGURE 5:**
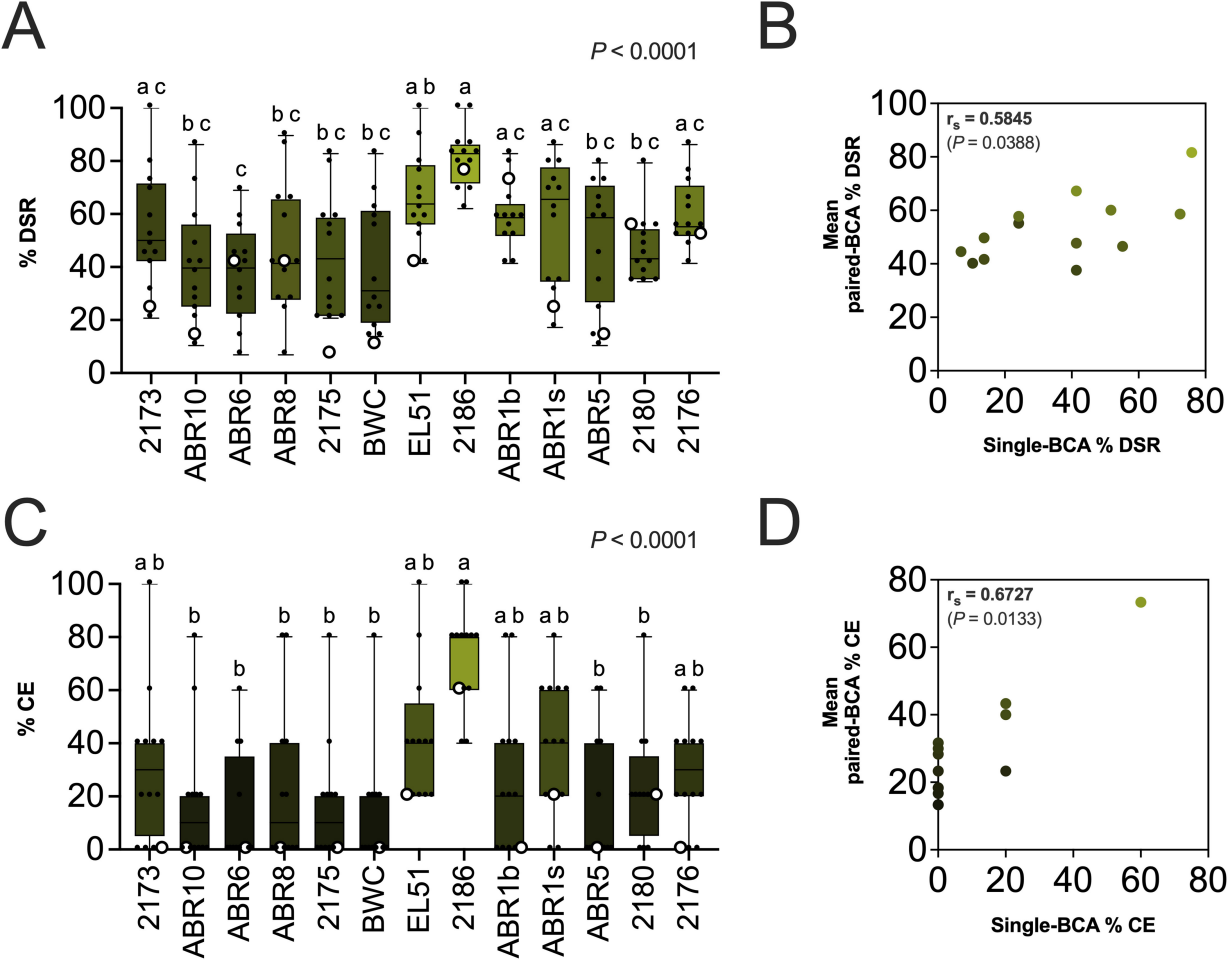
Suppression of fire blight symptoms on immature “Pink Lady” apples by individual and paired BCAs. **(A,C)** Percentage of disease-severity reduction (%DSR) **(A)** and control efficacy (%CE) values **(C)** for each BCA applied alone (white circles) or as part of a pairwise BCA combination (black dots). Each dot is an average value of 5 fruits. A value of 0% indicates symptoms equal to the pathogen-only control, while 100% indicates complete absence of symptoms. Single-BCA data superposed to pairwise treatments are shown for reference but were not included in the statistical analysis. Different letters indicate statistically significant differences among treatments (α = 0.05) **(B,D)** Spearman’s rank correlation between individual BCA efficacy and mean pairwise treatment efficacy. For each BCA, its individual %DSR **(B)** or %CE **(D)** (*x*-axis) was plotted against the mean %DSR or %CE of all pairwise combinations that included that specific BCA (*y*-axis). r_s_, represents Spearman’s rank correlation coefficient, and the *P*-value, the significance of the correlation. *Microbacterium* sp. 2173; *Microbacterium* sp. ABR10, *Priestia* sp. ABR6, *Curtobacterium* sp. ABR8; *Bacillus* sp. 2175, *Bacillus* sp. BWC; *Rahnella* sp. EL51, *Erwinia* sp. 2186; *Pantoea* sp. ABR1b, *Pantoea* sp. ABR1s, *Pseudomonas* sp. ABR5, *Pseudomonas* sp. 2180; yeast: *Rhodotorula* sp. 2176.

Pairwise BCA treatments enhanced fire blight suppression on immature “Pink Lady” apples as measured by %DSR and %CE at 14 dpi (Figure 5). The %DSR values for paired-BCA treatments ranged from 6.9% to 100%, with some combinations surpassing the efficacy of any single-BCA treatment (Figure 5 and Table 2). Approximately 57.6% of pairwise treatments reduced disease symptoms by 50% or more, and 11.5% paired treatments achieved %DSR values equal or above 80%, with none of the single-BCA treatments reaching the latter threshold (Figure 5A). Similarly, analysis of %CE values revealed that 21.8% paired-BCA treatments resulted in %CE values of 50% or above, and 12.8% paired treatments conferred 80% or more %CE, protection levels unattained by any single-BCA treatment (Figure 5B).

**TABLE 2:** Top pairwise isolate combinations with the highest disease control in fruit in terms of percentage disease reduction (%DSR) and control efficacy (%CE).

Single-isolate treatment^a^			Single-isolate treatment^a^			Paired-isolate treatment		
Isolate 1	%DSR	%CE	Isolate 2	%DSR	%CE	Mix (1 + 2)	%DSR	%CE
EL51	41%	20%	2186	75.9%	60%	EL51 + 2186	100.0%	100%
			ABR8	41.4%	0%	EL51 + ABR8	89.7%	80%
			2176	51.7%	0%	EL51 + 2176	75.9%	60%
2186	76%	60%	2173	24.1%	0%	2186 + 2173	100.0%	100%
			ABR10	13.8%	0%	2186 + ABR10	86.2%	80%
			ABR8	41.4%	0%	2186 + ABR8	86.2%	80%
			2175	6.9%	0%	2186 + 2175	82.8%	80%
			BWC	10.3%	0%	2186 + BWC	82.8%	80%
			ABR1b	72.4%	0%	2186 + ABR1b	82.8%	80%
			2180	55.2%	20%	2186 + 2180	79.3%	80%
			ABR5	13.8%	0%	2186 + ABR5	79.3%	60%
			ABR6	41.4%	0%	2186 + ABR6	69.0%	60%
ABR1s	24%	20%	ABR1b	72.4%	0%	ABR1s + ABR1b	79.3%	80%
			2176	51.7%	0%	ABR1s + 2176	86.2%	60%
			ABR5	13.8%	0%	ABR1s + ABR5	62.1%	60%

^a^*Rahnella* sp. EL51, *Erwinia* sp. 2186, *Pantoea* sp. ABR1b, *Pantoea* sp. ABR1s, *Pseudomonas* sp. 2180, *Pseudomonas* sp. ABR5, *Curtobacterium* sp. ABR8, *Microbacterium* sp. 2173, *Microbacterium* sp. ABR10, *Bacillus* sp. 2175, *Bacillus* sp. BWC, *Priestia* sp. ABR6, *Rhodotorula* sp. 2176.

The highest %DSR values were observed in paired treatments containing *Erwinia* sp. 2186, followed by *Pantoea* sp. ABR1s, *Rahnella* sp. EL51 and *Pantoea* sp. ABR1b (Figure 5A and Table 2). The analysis indicated a moderate positive correlation between single-BCA %DSR values and the average performance of the same BCA with others (r_*s*_ = 0.5845, *P* = 0.0388) (Figure 5B). In terms of %CE, the most effective paired treatments involved *Erwinia* sp. 2186, *Rahnella* sp. EL51 and *Pantoea* sp. ABR1s (Figure 5C and Table 2). However, in contrast to %DSR, results indicated a stronger positive monotonic relationship between single-BCA %CEs and the average %CE of paired treatments containing that BCA (r_*s*_ = 0.6727, *P* = 0.0133) (Figure 5D).

### 3.7 Net interactive behavior of paired BCAs

**FIGURE 6:**
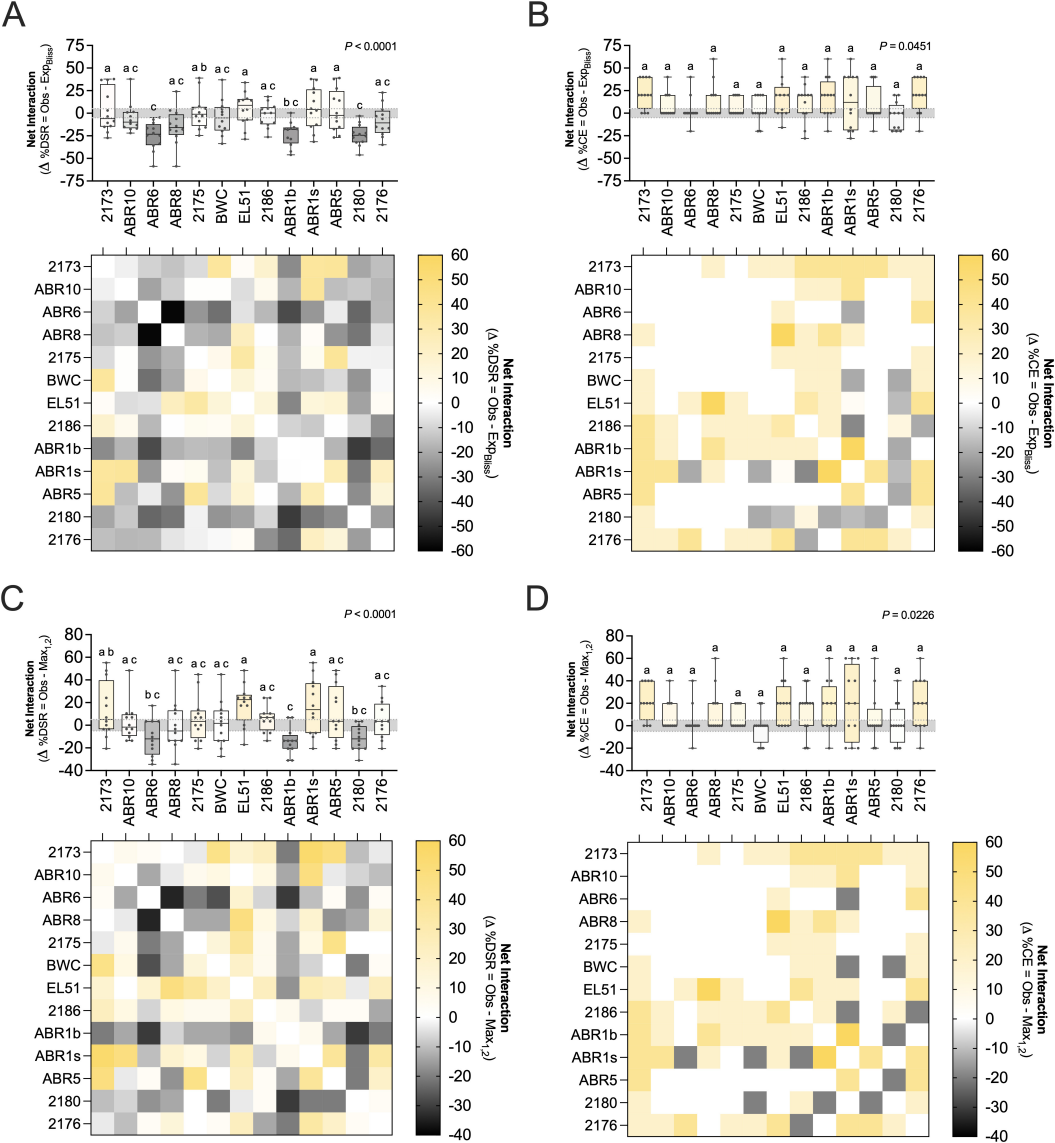
Net interaction effects of paired-BCA treatments in symptom development on immature “Pink Lady” apples. Box-and-whisker plots show the distribution of net interaction values for each strain (on the *x*-axis) combined with the remaining strains (gray dots). **(A,B)** Net interactions between strains calculated as the difference between the observed efficacy of the pair, assessed as percent disease severity reduction (%DSR) **(A)** or percent control efficacy (%CE) **(B)**, and the expected efficacy based on Bliss independence model with overlapping. **(C,D)** Net interactions between strains calculated as the difference between the observed efficacy of the pair, assessed as percent disease severity reduction (%DSR) **(C)** or percent control efficacy (%CE) **(D)**, and the highest of the two single-strain efficacies (best single agent criterium). Box plots show the median, interquartile range and range, with points indicating paired-strain net interaction values. The gray area between 5% and –5% indicates neutrality/additivity (no interaction). Different letters indicate statistically significant differences between groups based on *post-hoc* tests (*a* = 0.05). Heatmaps illustrate net biocontrol efficacy patterns for each pairwise combination. In the color scale, intense yellow indicates synergy (symptom suppression above the expected), white indicates neutral/additive interactions and black denotes antagonistic interactions effects (observed lesions larger than expected). *Microbacterium* sp. 2173; *Microbacterium* sp. ABR10, *Priestia* sp. ABR6, *Curtobacterium* sp. ABR8; *Bacillus* sp. 2175, *Bacillus* sp. BWC; *Rahnella* sp. EL51, *Erwinia* sp. 2186; *Pantoea* sp. ABR1b, *Pantoea* sp. ABR1s, *Pseudomonas* sp. ABR5, *Pseudomonas* sp. 2180; yeast: *Rhodotorula* sp. 2176.

Net interaction analysis allowed the identification of neutral, synergistic and antagonistic effects of BCA pairings on symptom development (Figure 6, Table 3, and Supplementary Figure 4). Under the Bliss independence framework, most BCA combinations impacted symptom severity (DSR%) antagonistically, indicating that the resulting disease suppression was often reduced compared to the expected effect if the two BCAs acted independently (Figure 6A and Table 3). An exception was paired treatments with *Rahnella* sp. EL51, which showed a moderately synergistic median Δ %DSR of 8.80% (Figure 6A and Table 3). In contrast, *Priestia* sp. ABR6 and *Pseudomonas* sp. 2180 formed antagonistic interactions with most BCAs, resulting in highly antagonistic median Δ %DSR values of −23.2% and −24.3%, respectively (Figure 6A and Table 3).

**TABLE 3:** Median net effects and frequency of synergistic, neutral and antagonistic disease suppression (assessed as %DSR and %CE) by paired BCA treatments in “Pink Lady” apples.

Model and BCA in pair^a^	Median Δ %DSR	Median net interaction behavior^b^	Frequency of interaction type (no. of pairs)^c^					Model and BCA in pair^a^	Median Δ %CE	Median net interaction behavior^b^	Frequency of interaction type (no. of pairs)^c^				
			Synergy		Neutr./Add.	Antagonism					Synergy		Neutr./Add.	Antagonism	
			High	Moderate	Indep.	Moderate	High				High	Moderate	Indep.	Moderate	High
Bliss independence								Bliss independence							
EL51	8.8	M. SYN	2	4	2	2	2	2173	20	H. SYN	9	0	3	0	0
ABR1s	4.1	N/ADD	4	2	2	2	2	2176	20	H. SYN	9	0	2	0	1
2186	0.2	N/ADD	0	4	3	4	1	EL51	20	H. SYN	8	0	3	0	1
2175	−1.1	N/ADD	2	3	3	3	1	ABR1b	20	H. SYN	7	0	4	1	0
ABR5	−2.3	N/ADD	3	2	2	3	2	2186	20	H. SYN	7	1	2	0	2
BWC	−5.2	M. ANT	1	2	3	4	2	ABR1s	12	M. SYN	6	0	2	1	3
2173	−6.1	M. ANT	3	1	2	4	2	2175	0	N/ADD	5	0	7	0	0
ABR10	−9.8	M. ANT	1	1	3	6	1	ABR5	0	N/ADD	4	0	8	0	0
2176	−10.6	M. ANT	1	1	3	4	3	ABR8	0	N/ADD	4	0	6	0	2
ABR8	−16.0	M. ANT	1	0	2	6	3	BWC	0	N/ADD	4	0	8	0	0
ABR1b	−18.0	M. ANT	0	0	1	6	5	ABR10	0	N/ADD	3	0	8	0	1
ABR6	−23.2	H. ANT	0	0	1	4	7	ABR6	0	N/ADD	2	0	9	0	1
2180	−24.3	H. ANT	0	0	1	2	9	2180	0	N/ADD	2	1	4	2	3
**Best single agent**								**Best single agent**							
EL51	22.4	H. SYN	7	2	2	1	0	2173	20	H. SYN	9	0	3	0	0
ABR1s	13.8	M. SYN	5	3	0	3	1	EL51	20	H. SYN	8	0	4	0	0
2186	6.9	M. SYN	2	5	2	3	0	2176	20	H. SYN	8	0	3	0	1
2173	5.2	M. SYN	4	2	4	1	1	2186	20	H. SYN	8	0	2	0	2
2176	3.5	N/ADD	3	1	5	2	1	ABR1s	20	H. SYN	7	0	2	0	3
ABR5	3.5	N/ADD	5	0	4	2	1	ABR1b	20	H. SYN	7	0	4	0	1
2175	3.5	N/ADD	2	4	3	2	1	ABR8	0	N/ADD	5	0	7	0	0
BWC	1.7	N/ADD	2	3	2	3	2	2175	0	N/ADD	4	0	8	0	0
ABR10	−1.7	N/ADD	1	4	3	4	0	ABR10	0	N/ADD	4	0	8	0	0
ABR8	−5.2	M. ANT	1	3	2	5	1	ABR5	0	N/ADD	3	0	7	0	2
2180	−12.1	M. ANT	0	0	4	4	4	2180	0	N/ADD	3	0	6	0	3
ABR6	−12.1	M. ANT	0	1	3	4	4	BWC	0	N/ADD	2	0	7	0	3
ABR1b	−13.8	M. ANT	0	2	0	6	4	ABR6	0	N/ADD	2	0	9	0	1

^a^*Rahnella* sp. EL51, *Erwinia* sp. 2186, *Pantoea* sp. ABR1b, *Pantoea* sp. ABR1s, *Pseudomonas* sp. ABR5, *Pseudomonas* sp. 2180, *Priestia* sp. ABR6, *Bacillus* sp. 2175, *Bacillus* sp. BWC, *Curtobacterium* sp. ABR8, *Microbacterium* sp. 2173, *Microbacterium* sp. ABR10.

^b^Categories based on median net Δ %DSR and Δ %CE values: H. SYN, highly synergistic (Δ ≥ 20%); M. SYN, moderately synergistic (5.0% ≤ Δ < 20.0%); N/ADD, Neutral/Additive (−5% < Δ < 5%); M. ANT, moderately antagonistic (−20.0% < Δ ≤ −5.0%); H. ANT, highly antagonistic (Δ ≤ −20.0%).

^c^Number of paired-BCA combinations (out of 12) for which a specific BCA exhibited the net interaction category indicated in the column header.

Net interaction analysis based on %CE revealed a different pattern, where most pairwise BCA treatments had neutral/additive or synergistic effects on disease suppression (Figure 6B and Table 3). BCA pairings with *Microbacterium* sp. 2173, *Rhodotorula* sp. 2176, *Rahnella* sp. EL51, *Pantoea* sp. ABR1b and *Erwinia* sp. 2186 were classified as highly synergistic, with median Δ %CE values of 20%. Paired treatments with *Pantoea* sp. ABR1s had moderate synergistic effects on symptom suppression (median Δ %CE of 12%), while treatments involving the remaining 7 BCAs were classified as neutral (Table 3). For several BCAs (2173, EL51, ABR1b), the strong synergy detected based on Δ %CE under the Bliss independence model contrasted with antagonistic or neutral effects measured by Δ %DSR (Table 3).

Analysis under the “best single agent” framework revealed similar trends for %CE (Figures 6C,D and Table 3). For %DSR, however, synergistic effects were generally amplified compared to calculations under the Bliss independence model. As an example, the median Δ %DSR for *Rahnella* sp. EL51 increased from moderately synergistic 8.8% (Bliss) to highly synergistic 22.4% (best single agent), and several other BCAs (*Pantoea* sp. ABR1s, *Erwinia* sp. 2186, *Microbacterium* sp. 2173) shifted from neutral or antagonistic to synergistic categories (Figures 6A,B and Table 3).

### 3.8 Population dynamics associated with symptoms suppression by *Erwinia* sp. 2186 and *Rahnella* sp. EL51 in immature apple fruitlets

**FIGURE 7:**
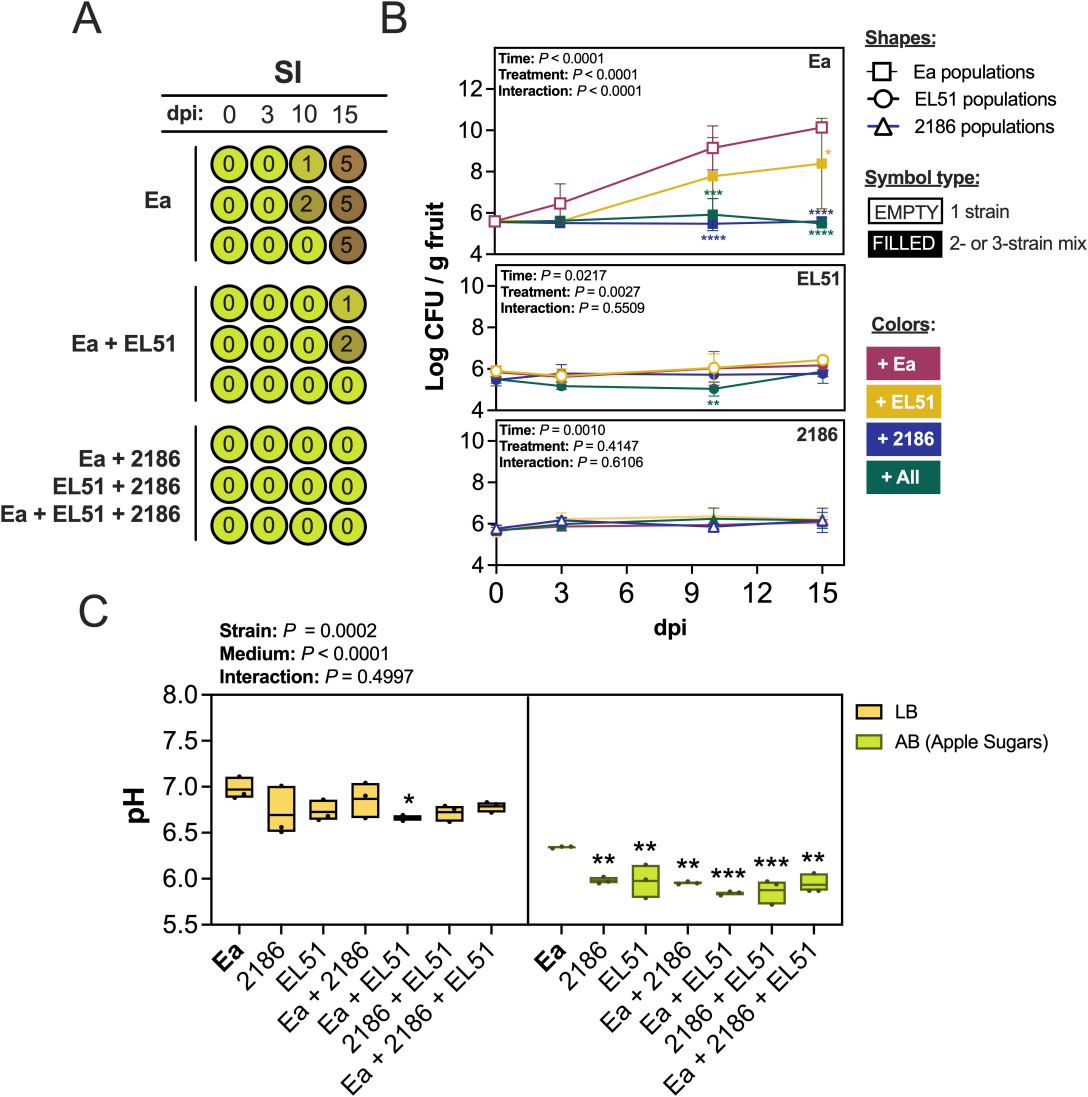
Interplay among *Erwinia amylovora* Z2274^Rif^ (Ea) and two antagonists (*Rahnella* sp. EL51^Nal^ and *Erwinia* sp. 2186) in immature “Pink Lady” apples. **(A)** Schematic timeline of symptom development. Each circle represents one apple replicate. Numbers within each circle are symptom ratings based on a severity index (SI) where 0 means no symptoms and 6, fully necrosed fruit. **(B)** Population dynamics in detached fruit. Lines show the mean Log-transformed CFU/gram of fruit ± standard deviation. The summary of a two-way ANOVA analysis of each data set is detailed on the upper left corner of each chart. **(C)** Effect of strain growth on medium acidification. The pH of the medium of three replicates is represented with box plots for each treatment. The significancy of medium, treatment (strain combination) and interaction effects on medium pH is summarized on the upper left side of the chart. Asterisks indicate significant differences with Ea alone at the same time point **(B)** or grown in the same medium **(C)**: *P < 0.05; **P < 0.01; ***P < 0.001; ****P < 0.0001.

To investigate the interaction between *E. amylovora* and the two top-performing BCAs, *Rahnella* sp. EL51 and *Erwinia* sp. 2186, we monitored symptom progression and bacterial population dynamics over time on immature apple fruit (Figures 7A,B) and investigated effects of BCA growth in medium pH acidification (Figure 7C).

Preventive application of *Rahnella* sp. EL51, *Erwinia* sp. 2186 or a mix of both strains 24 h before *E. amylovora* challenge strongly suppressed symptom development over 15 days (Figure 7A). While apples inoculated with *E. amylovora* alone reached an average SI of 5 by 15 dpi, all BCA treatments, whether single or combined, led to minimal or no symptoms (mean SI ≤ 1). In the absence of BCAs, *E. amylovora* populations increased steadily, reaching around 10 Log CFU/g by 15 dpi, with visual detection of symptom development coinciding with pathogen populations exceeding 8 Log CFU/g (Figures 7A,B). All BCA treatments significantly reduced *E. amylovora* populations at 10–15 dpi (*P* ≤ 0.0390). By 15 dpi, mean pathogen counts in apples treated with *Rahnella* sp. EL51, *Erwinia* sp. 2186 or both were 8.4, 5.6 and 5.5 Log CFU/g, respectively (Figure 7B).

Populations of both BCAs alone and in *E. amylovora*-challenged fruit, remained stable over time at approximately 6 Log CFU/g. A slight but significant reduction in *Rahnella* sp. EL51 density (to 5.0 Log CFU/g at 10 dpi) was observed only in the paired-BCA treatment with *Erwinia* sp. 2186 of fruit challenged with *E. amylovora* (Figure 7B).

To assess whether growth inhibition of *E. amylovora* was linked to medium acidification, we measured pH in rich (LB) and minimal (AB) media, the latter supplemented with carbohydrates reflecting immature apple composition (Figure 7C). In LB, pH remained near neutral (around pH 6.8) for most treatments. Only co-cultures of *Rahnella* sp. EL51 and *E. amylovora* caused a slight but significant decrease to pH 6.7 (Figure 7C). In contrast, in AB medium (pH 6.8), all treatments containing BCAs significantly acidified the medium to approximately pH 5.9 (*P* ≤ 0.0020), with the lowest value (pH 5.8) occurring in EL51 and *E. amylovora* co-cultures (*P* ≤ 0.0002).

## 4 Discussion

Fire blight-infected apple tissues are host of microbial species that compete and cooperate to explore shared niche resources. Under the hypothesis that the shared niche may lead to the selection of microorganisms able to suppress or restrict *E. amylovora* growth, we evaluated the potential of a collection of isolates as BCAs, in alignment with prior successes in isolating potent BCAs from diseased plants against different pathogens (Anees et al., 2010; Biosca et al., 2024; Huang et al., 2013; Tolba and Soliman, 2013). The selected isolates belonged to genera such as *Pantoea*, *Microbacterium*, *Bacillus*, *Erwinia*, *Curtobacterium* and *Rahnella*, previously associated with healthy and diseased *E. amylovora* hosts (Barbé et al., 2022; Pusey et al., 2009; Steven et al., 2018; Wallis et al., 2021). Furthermore, many species belonging to these genera have previously been reported to have antagonistic activity against *E. amylovora* (Abo-Elyousr et al., 2011; Bonaterra et al., 2022; Cui et al., 2023; Esteban-Herrero et al., 2023; Kong et al., 2021; Pusey et al., 2009; Stockwell et al., 2010; Sundin et al., 2009). All the BCA candidates produced siderophores and at least one hydrolytic exoenzyme potentially associated with their biocontrol activities (Bonaterra et al., 2022; Saberi Riseh et al., 2024; Xie et al., 2024).

Results in our study demonstrated that antagonism detection by an agar plug assay was medium-dependent. While minimal media (AB, M9) produced sharper inhibition zones, nutrient-rich media (LA or KBA) did not. In this regard, supplementing AB medium with yeast extract neutralized inhibition zone formation, directly linking these results with those on LA. This pattern is consistent with antimetabolite-mediated antagonism, where rich media components such as yeast extract supply the metabolites, whose biosynthesis is targeted by the antimetabolite toxin, thus rescuing pathogen growth (Arrebola et al., 2011; Gasson, 1980; Wallace et al., 2020).

Carbon source composition also significantly impacted *in vitro* antagonism. Addition of sugars like glucose, fructose, sucrose and glycerol supported pathogen inhibition in over 50% of tested isolates. In contrast, sorbitol rarely elicited antagonistic activity *in vitro*, despite being the predominant transport carbohydrate in apple and pear trees (Pleyerová et al., 2022) and a main component in immature apples and pears (Larson et al., 2023; Yin et al., 2024). This disparity may reflect metabolic trade-offs, as sorbitol utilization is energetically demanding and may repress secondary metabolite production critical for antagonism. Interestingly, among the few isolates showing certain *in vitro* antagonistic activity on AB plus sorbitol (*Bacillus* sp. strains 2175 and BWC, *Erwinia* sp. 2186 and *Pseudomonas* sp. ABR5), only *Erwinia* sp. 2186 and *Pseudomonas* sp. ABR5 provided significant disease control in fruitlets. These results suggest that carbon catabolite repression and other regulatory mechanisms, shaped by the metabolite composition of fruitlets and interspecies metabolic interactions, might govern microbial competition in these environments (Park et al., 2020; Reding, 2022; Ruiz-Villafán et al., 2022). Within the fruit, carbon source composition and distribution are spatially and temporally dynamic, which also likely explains discrepancies between the observed *in vitro* and *in vivo* biocontrol efficacies (Braun and Hildebrand, 2005; Gu et al., 2021; Larson et al., 2023; Lei et al., 2025; Li et al., 2012, 2018; Pleyerová et al., 2022; Watari et al., 2004; Yin et al., 2024).

Physico-chemical parameters, such as pH and temperature, also modulated *in vitro* biocontrol efficacy as demonstrated previously (Bosmans et al., 2016; Dickie and Bell, 1995; Gasson, 1980; Sipiczki, 2023; Whipps, 1987). Slightly acidic conditions (pH 6.0) and lower temperatures (22°C) consistently enhanced *in vitro E. amylovora* inhibition across Gram-positive and Gram-negative BCAs, highlighting how environmental variability (fluctuations in temperature, humidity, apoplastic pH, carbon source availability) can constrain biocontrol performance *in planta*, across distinct field trials, and environmental conditions (Bonaterra et al., 2012, 2022; DuPont et al., 2023; Harms et al., 2021; van der Zwet et al., 2016a). Thus, optimization of BCA pre-culture conditions before field applications might improve and enhance the robustness and efficacy of *in vivo* BCA application (Cabrefiga et al., 2011; Stockwell et al., 1998).

As a systemic pathogen, *E. amylovora* rapidly invades host tissues from initial infection sites, making the timing of BCA application critical for effective disease management. In *ex vivo* assays with immature pear fruitlets, preventive treatments outperformed BCA-pathogen co-inoculations, especially with Gram-negative BCAs, indicating the importance of early niche occupation, resource preemption, and/or production of inhibitory compounds to restrict or inhibit the establishment of *E. amylovora* populations (Cabrefiga et al., 2011; Pusey et al., 2009; Stockwell et al., 2010, 2013; Sundin et al., 2009; Zeng et al., 2023).

A higher number of single-BCA treatments delayed the onset of symptoms, reduced symptom severity and/or achieved complete disease suppression in apples compared to pears. These host-dependent infection dynamics may be related to the better adaptation of the tested organisms to apple (the host they were isolated from), as well as to a broader temporal window for biocontrol action. Gram-negatives generally exhibited better biocontrol efficacy than Gram-positives, probably due to metabolic similarity with *E. amylovora* (Russel et al., 2017; Schlechter et al., 2023). In this regard, although we did not characterize the metabolic profile of the isolates, all Gram-negatives were able to use sorbitol, glucose, sucrose, fructose and glycerol as carbon sources in minimal media, and many of them were able to grow on *E. amylovora* selective CCT medium, indicating similar tolerance to high osmolyte concentration (10% sucrose and 1% sorbitol) and resistance to selective components such as thallium nitrate, tergitol 7, and crystal violet. Besides, *Rahnella* sp. EL51, *Erwinia* sp. 2186, *Pantoea* sp. ABR1b and *Pantoea* sp. ABR1s belonged to the same order or family as *E. amylovora* (order Enterobacteriales, family Erwiniaceae), which often correlate with common metabolic capabilities (Li et al., 2023).

Biocontrol agent combinations improved disease control, with many paired treatments surpassing 60% CE. Notably, the most effective combinations (*Erwinia* sp. 2186 paired with either *Rahnella* sp. EL51 or *Microbacterium* sp. 2173) completely prevented symptom development in immature apples. BCA and pathogen population dynamics in apple fruitlets revealed that symptom reduction by EL51 + 2186 treatment was associated with *E. amylovora* growth inhibition without an apparent decline of viable cells below initial levels. In fact, over a 15-days period, neither *E. amylovora* nor *Rahnella* sp. EL51 and *Erwinia* sp. 2186 populations changed significantly, supporting a competition-based interaction and niche exclusion as the dominant mode of action, potentially augmented by other non-lethal processes like environmental modification (pH acidification) or induction of host defenses.

Although most pairwise combinations resulting in high protection levels involved at least one isolate that alone, provided intermediate-to-high protection (*Erwinia* sp. 2186), this was not a rule for all efficacious combinations. In some cases, isolates that when applied alone did not achieve significant protection levels resulted in moderate-high disease suppression when combined (*Pantoea* sp. ABR1s and *Pseudomonas* sp. ABR5). Experimental and modeling studies indicate that disease control efficacy of BCA mixture is dependent on biocontrol mechanisms involved, spatial heterogeneity, stability and survival of the BCA mixture and incompatibilities between the BCAs involved (Bonaterra et al., 2012, 2022; Nunes et al., 2024; Xu et al., 2011; Xu and Jeger, 2013b). In our study, all isolates produced siderophores, and most of them secreted, at least, one hydrolytic exoenzyme, which could complement the biocontrol activity in the mix.

Most studies on biocontrol using microbial consortia fail to quantify interactions between constituent BCAs. We tested for additive, synergistic or antagonistic interactions using both the Bliss independence and “best single agent” frameworks. The independence/non-interference condition of the Bliss model defines the baseline for detecting synergism or antagonism. However, it is difficult to imagine two microorganisms occupying the same niche without some level of interference (such as competition for resources and space). This suggests that the theoretical Bliss baseline may be biologically unachievable, and may explain, at least in part, our results. In our study, similar to previous reports (Xu et al., 2011; Xu and Jeger, 2013a), synergistic interactions leading to high net Δ %DSR were rare, with most pairwise treatments showing neutral or antagonistic effects. The trend toward antagonism detected by the model may be linked, at least in part, to unavoidable ecological interference between the BCAs. Interestingly, the frequency of synergisms increased when Δ %DSR data were analyzed under the “best single agent” framework compared to Bliss. This might indicate that, while undesired BCA interactions such as competition for resources and space likely prevented most combinations from reaching their theoretical independent potential for reducing symptom severity, the mixtures still performed as well as or better than the best single BCA of the pair. This pattern of “best single agent” neutrality or synergy indicates that, despite ecological interference, many combinations retained potential utility for disease management.

In contrast to %DSR-based results, when %CE was used to assess the net efficacy of paired-BCA treatments (D%CE), most net interactions were neutral or synergistic regardless of the model used. Both parameters, capture different phases of *E. amylovora* infection: %CE is linked to the pathogen’s success in establishing initial infection, whereas %DSR characterizes the subsequent progression of symptom development. Our data indicate that paired-BCA treatments either had neutral impact on *E. amylovora* establishment (performing similarly as individual BCAs) or contributed synergistically to blocking initial infection. While the Bliss model’s sensitivity to low baseline effects may have contributed to the frequency of observed synergies for %CE, the consistency of this pattern with those obtained using the “best single agent” framework likely indicates a genuine tendency of paired treatments to synergistically block infection establishment. These results align with those of Cui et al. (2021) on flower stigmas, suggesting that BCA mixes tend to enhance niche exclusion and prevent pathogen establishment at the wound sitethereby reducing disease incidence. However, the neutral or antagonistic effects observed for %DSR in the same combinations suggest that once the pathogen overcomes this initial barrier, inter-BCA competition within infected tissues may compromise individual antagonistic activities or alter the host environment to the pathogen’s advantage, resulting in predominantly antagonistic net interactions when calculated using %DSR.

Based on our data, synergistic interactions were strain-dependent, aligning with reports by others (Cui et al., 2021). Our paired-treatment analysis revealed the existence of “BCA helper” microorganisms like *Rahnella* sp. EL51, which interactions with other microorganisms were mostly beneficial and tended to enhance biocontrol efficacy synergistically. In contrast, there were other microorganisms like *Pseudomonas* sp. 2180 or *Priestia* sp. ABR6, which mostly interacted antagonistically with other BCAs. These results suggest that cultural practices enriching the orchard phyllosphere with “BCA helper” organisms like *Rahnella* sp. EL51 or reducing populations of “BCA hinderer” organisms like *Pseudomonas* sp. 2180 may improve the efficacy of commercial BCAs against fire blight and other diseases as proposed by Li et al. (2022).

Overall, our study highlights the ecological and metabolic complexity underlying microbial antagonism against *E. amylovora* and confirms symptomatic plant material as a promising source of BCAs for disease control. Key factors influencing *in vitro E. amylovora* growth inhibition included carbon source, media pH and incubation conditions. *Ex vivo* assays also revealed host-related differences in biocontrol efficacy as well as the importance of BCA application timing. Furthermore, while single-BCA treatments showed variable success in disease suppression, strategic pairwise combinations enhanced disease control. Our approach also allowed us to identify strain-dependent “helper” and “hinderer” behaviors that would have been missed by conventional analyses. As in previous reports (Besset-Manzoni et al., 2019), our results demonstrate that BCA selection exclusively based on *in vitro* tests may lead to choosing highly incompatible BCA partners for experiments *in vivo*. However, further validation under field conditions is required to determine the significance of protection provided by BCA treatments, particularly in comparison with conventional antibiotics and copper. Dissecting the mechanisms behind strain interactions and how these translate to disease suppressionare also critical. Integrating these insights with existing control strategies could pave the way for more robust and sustainable fire blight management.

## Data Availability

The original contributions presented in the study are publicly available. This data can be found here: NCBI accession numbers: PZ173380 (*Microbacterium* sp. 2173), PZ173381 (*Microbacterium* sp. ABR10), PZ173382 (*Curtobacterium* sp. ABR8), PZ173383 (*Priestia* sp. ABR6), PZ173384 (*Bacillus* sp. 2175), PZ173385 (*Bacillus* sp. BWC), PZ173386 (*Rahnella* sp. EL51), PZ173387 (*Erwinia* sp. 2186), PZ173388 (*Pantoea* sp. ABR1s), PZ173389 (*Pantoea* sp. ABR1b), PZ173390 (*Pseudomonas* sp. ABR5), PZ173391 (*Pseudomonas* sp. 2180), and PZ173542 (*Rhodotorula* sp. 2176).
