# Supplementary material for: Environmental conditions and ecological interactions among microorganisms isolated from diseased samples shape their biocontrol efficacy against Erwinia amylovora
Source: Front Microbiol. 2026 Apr 10;17:1816023. doi: 10.3389/fmicb.2026.1816023 (PMC13106295; doi:10.3389/fmicb.2026.1816023)
Supplement: Supplementary file 1 [file Data_Sheet_1.pdf]

Environmental Conditions and Ecological Interactions Among Saprophytes Isolated from Diseased Samples Shape Their Biocontrol Efficacy Against *Erwinia amylovora*

Ricardo Delgado Santander, Youfu Zhao*

Department of Plant Pathology, College of Agricultural, Human, and Natural Resource Sciences, Irrigated Agriculture Research and Extension Center, Washington State University, Prosser, WA, U.S.A.

*** Correspondence:**
Youfu Zhao
youfu.zhao@wsu.edu

Supplementary Figures

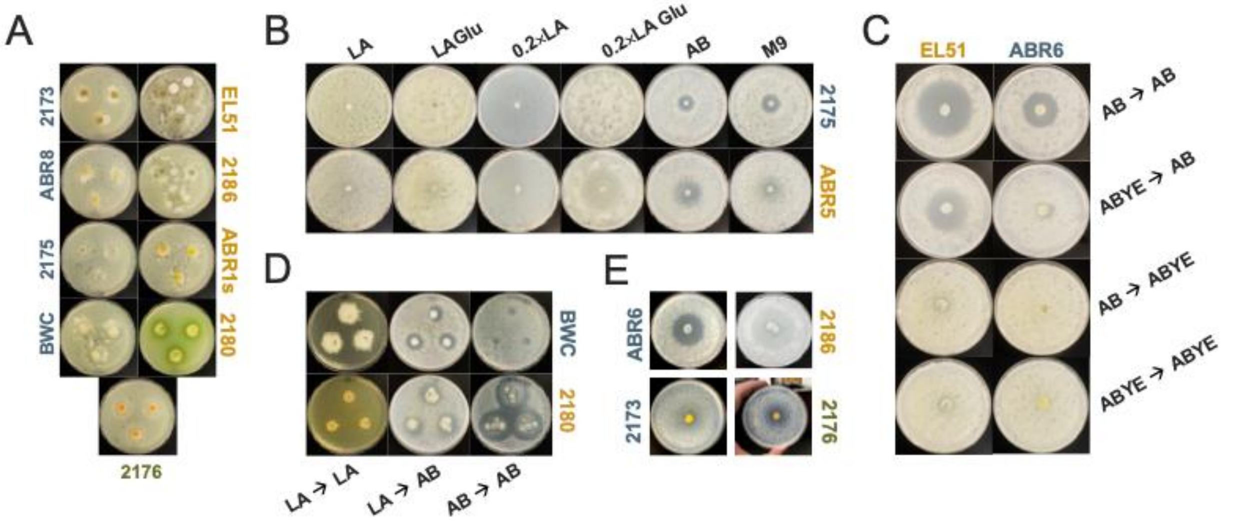

Supplementary Figure S1. Optimization of an agar plug assay for quantification of *E. amylovora* growth inhibition by saprophytes. (A) Saprophytes grown on and transferred to KBA plates. (B) Saprophytes grown on LA and transferred to: LA; LA amended with 0.4% glucose (LAGlu); 0.2× diluted LA (0.2×LA); 0.2×LA plus 0.4% glucose (0.2×LAGlu); minimal AB agar with 0.4% glucose and 0.05% nicotinic acid (AB); and minimal M9 agar amended with 0.4% Glucose and nicotinic acid (M9). (C) Saprophytes grown on AB or AB with 0.5% yeast extract (ABYE) and transferred to AB or ABYE. (D) Enhanced inhibition zone detection in Gram-positives (top) when grown on LA and transferred to AB, and in Gram-negatives (bottom) when grown on AB and transferred to AB (bottom). (E) Main halo types observed in this study: Clearly defined zones with complete *E. amylovora* growth inhibition (top left); diffuse halos with defined margins, where a stepwise reduction in *E. amylovora* lawn density (top right); faint, diffuse halos with undefined margins (bottom left and right). Pictures taken after 16 h on rich media, and 72h on minimal media. Blue, yellow and green are used to distinguish Gram-positives (*Microbacterium* sp. 2173, *Curtobacterium* sp. ABR8, *Bacillus* sp. 2175, *Bacillus* sp. BWC, *Priestia* sp. ABR6), Gram-negatives (*Rahnella* sp. EL51, *Erwinia* sp. 2186, *Pantoea* sp. ABR1s, *Pseudomonas* sp. ABR5, *Pseudomonas* sp. 2180) and yeast (*Rhodotorula* sp. 2176), respectively. KBA: King's B agar; LA: Lysogeny agar; AB and M9: minimal medium AB and M9.

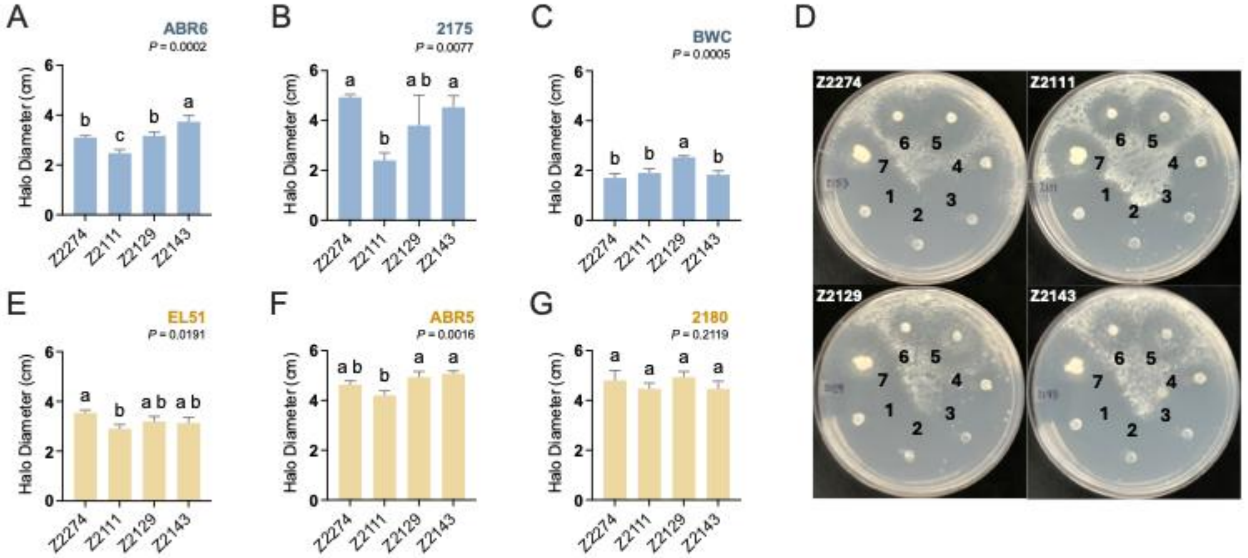

Supplementary Figure S2. Strain-dependent *E. amylovora* growth inhibition by different Gram-positive and Gram-negative bacterial antagonists. Antagonist plugs were transferred to minimal AB agar amended with 0.4% glucose and 0.05% nicotinic acid spread-plated with different *E. amylovora* strains from Washington, U.S.A.. Growth inhibition diameters were measured after 72h incubation at 22ºC. (A-F) Differential growth inhibition of representative *E. amylovora* strains by Gram-positive (A-C) and Gram-negative bacteria (E-G). *P* values indicate the significance of comparisons based on a one-way ANOVA, and different letters denote statistically significant differences between *E. amylovora* strains, based on Tukey's comparison tests (α = 0.05). (D) *In vitro* growth inhibition of different *E. amylovora* strains of the predominant Large Chromosomal Inversion-types from different locations in Washington (Yang et al., 2023) (Z2274, Ea1189-type; Z2111, CFBP1430-type; Z2129, Ea273-type; Z2143, Ea1189-type) by representative saprophytic bacterial antagonists from diseased apple tissue: 1, *Bacillus* sp. 2175; 2, *Pseudomonas* sp. 2180; 3, *Pseudomonas* sp. ABR5; 4, *Priestia* sp. ABR6; 5, *Pseudomonas* sp. 2180; 6, *Rahnella* sp. EL51; 7, *Bacillus* sp. BWC.

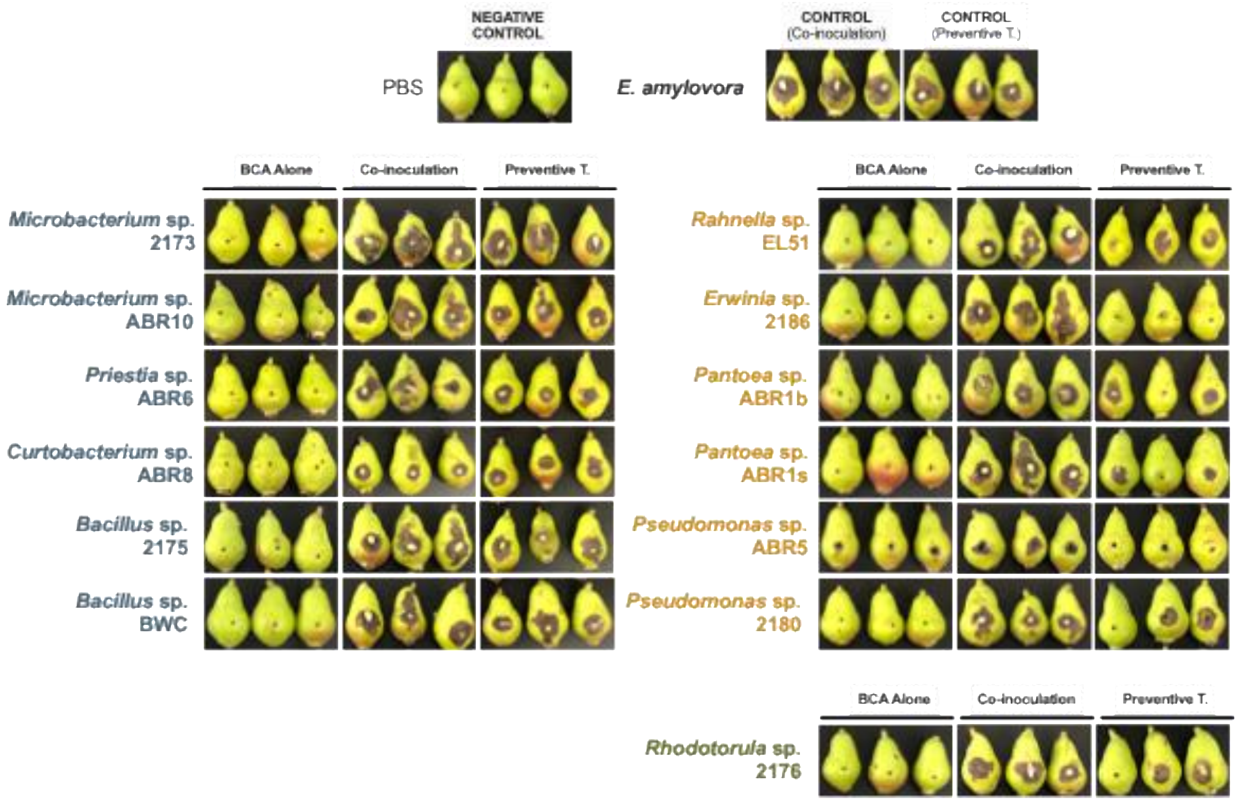

Supplementary Figure S3. Suppression of fire blight symptoms by antagonists in immature 'Bartlett' pears under co-inoculation and preventive treatment conditions. Symptoms were photographed 6 days post-*E. amylovora* inoculation. Pictures show representative results of 3 out of 6-7 fruits inoculated per treatment. Blue, yellow and green color fonts represent Gram-positive, Gram-negative and yeast BCAs, , respectively. BCA: bacterial biocontrol agent.

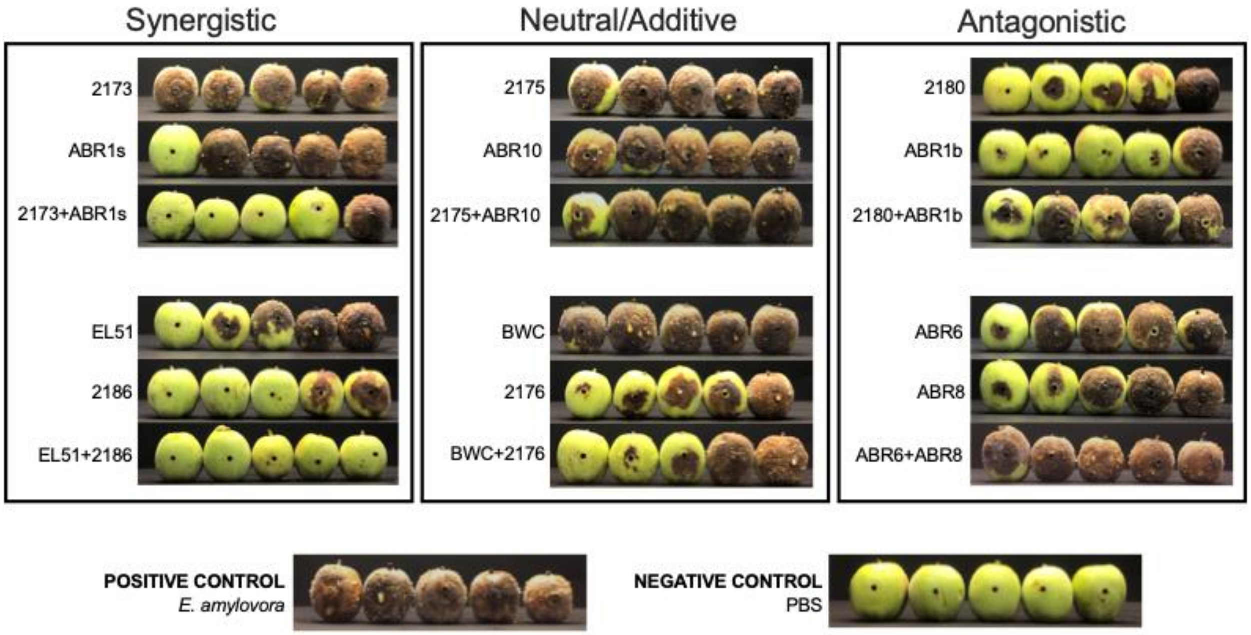

Supplementary Figure S4. Examples of synergistic, neutral/additive and antagonistic fire blight disease suppression by paired antagonist treatments in immature 'Pink Lady' apples. *Rahnella* sp. EL51, *Erwinia* sp. 2186, *Bacillus* sp. 2175, *Microbacterium* sp. ABR10, *Bacillus* sp. BWC, *Rhodotorula* sp. 2176, *Pseudomonas* sp. 2180, *Pantoea* sp. ABR1b, *Priestia* sp. ABR6, *Curtobacterium* sp. ABR8.

References

Yang, H.-W., Thapa, R., Johnson, K., DuPont, S.T., Khan, A., Zhao, Y., 2023. Examination of Large Chromosomal Inversions in the Genome of *Erwinia amylovora* Strains Reveals Worldwide Distribution and North America-Specific Types. Phytopathology 113, 2174–2186. https://doi.org/10.1094/PHYTO-01-23-0004-SA
